# Arrival and magnetization of carbonaceous chondrites in the asteroid belt before 4562 million years ago

**DOI:** 10.1038/s43247-020-00055-w

**Published:** 2020-12-04

**Authors:** Timothy O’Brien, John A. Tarduno, Atma Anand, Aleksey V. Smirnov, Eric G. Blackman, Jonathan Carroll-Nellenback, Alexander N. Krot

**Affiliations:** 1 Department of Earth and Environmental Sciences, University of Rochester, Rochester, NY 14627 USA; 2 Department of Physics and Astronomy, University of Rochester, Rochester, NY 14627 USA; 3 Department of Geological and Mining Engineering and Sciences, Michigan Technological University, Houghton, MI 49931 USA; 4 Physics Department, Michigan Technological University, Houghton, MI 49931 USA; 5 Hawai’i Institute of Geophysics and Planetology, University of Hawai’i at Manoa, Honolulu, HI 96822 USA

**Keywords:** Asteroids, comets and Kuiper belt, Meteoritics, Early solar system

## Abstract

Meteorite magnetizations can provide rare insight into early Solar System evolution. Such data take on new importance with recognition of the isotopic dichotomy between non-carbonaceous and carbonaceous meteorites, representing distinct inner and outer disk reservoirs, and the likelihood that parent body asteroids were once separated by Jupiter and subsequently mixed. The arrival time of these parent bodies into the main asteroid belt, however, has heretofore been unknown. Herein, we show that weak CV (Vigarano type) and CM (Mighei type) carbonaceous chondrite remanent magnetizations indicate acquisition by the solar wind 4.2 to 4.8 million years after Ca-Al-rich inclusion (CAI) formation at heliocentric distances of ~2–4 AU. These data thus indicate that the CV and CM parent asteroids had arrived near, or within, the orbital range of the present-day asteroid belt from the outer disk isotopic reservoir within the first 5 million years of Solar System history.

## Abstract (web summary)

Carbonaceous chondrite parent bodies entered the inner solar system earlier than 4562 million years ago and were magnetized by the solar wind rather than in differentiated objects, according to paleomagnetic analyses of Vigarano and Mighei type meteorites

## Introduction

The early migration of Jupiter is thought to have resulted in scattering of planetesimals and ultimately the diversity of meteorite types found in the asteroid belt today^1–3^. Magnetic minerals can be created and preexisting grains transformed during aqueous alteration, and such events occurred on the CM and CV parent bodies at ~4.8 and ~4.2 m.y. after CAI formation, respectively^4,5^. As we will show using theory and ideal magnetohydrodynamic simulations, magnetizations can be imparted on these minerals by the solar wind, and the recorded field strength can constrain for the first time the heliocentric distance of the parent asteroids at the time of aqueous alteration. However, meteorite magnetizations can also be of intrinsic origin, reflecting the physical nature of magnetic minerals rather than ambient fields^6^, or they can record past parent body core dynamos^7–9^. Prior magnetic work on the CV meteorite Allende has been used to argue for a core dynamo^10^ seemingly overturning more than 50 years of research that held this meteorite as a type example from an undifferentiated asteroid^11^. This paradox must be addressed first before using carbonaceous chondrite meteorite magnetizations to constrain early Solar System events.

Allende was long considered to be a primitive aggregate of CAIs, chondrules, and fine-grained matrix. Subsequently, it was recognized that CV chondrites had experienced fluid-assisted thermal metamorphism and metasomatic alteration resulting in the formation of secondary minerals^12^, including Fe-sulfides and magnetite important for magnetism. Allende (CV3.6) is one of the most metamorphosed CV chondrites, having experienced peak temperatures of ~500–600 °C^13^. Early work recognized that Allende has a magnetization^7^, but only recently has it been claimed that this requires a core dynamo^10^; an interpretation used by some authors as prima facie evidence for differentiation^14^. It is possible to construct thermal models such that CV chondrites originate in the outermost layers of a partially differentiated asteroid, isolated from interior melting^15^. But no meteorites are known that clearly represent mantle or core samples of the hypothetical differentiated CV asteroid. Achondrite and CV clasts mixed in a breccia are also unknown. Moreover, the estimated initial abundance of ^26^Al in the CV parent asteroid, considered to be its major heating source, is insufficient to produce melting and igneous differentiation^16^. Another model suggests that CV chondrites could represent a “late” accretion (~1.5 to 5 m.y. after CAI formation) to a body that had already differentiated^17^, but the lack of differentiated meteorites unambiguously linked to the CV parent asteroid remains an outstanding question.

## Results and discussion

**Fig. 1:**
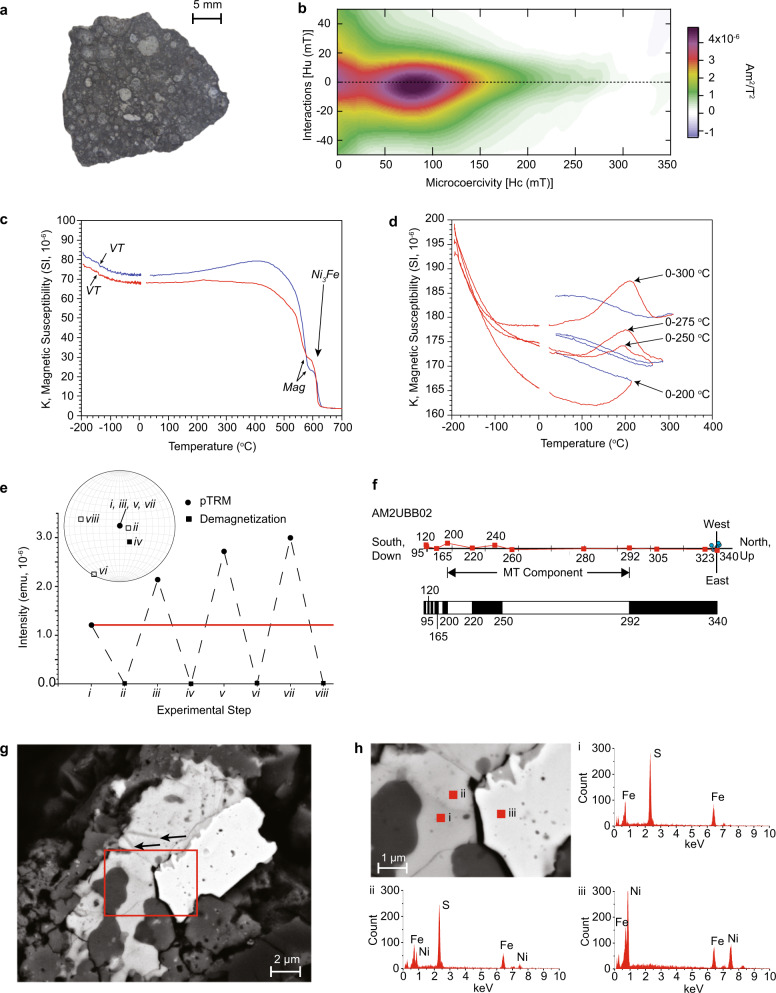
Rock magnetism, electron microscopy and tests of Allende paleomagnetic recording fidelity. **a** Typical Allende sample analyzed. **b** First-order reversal curve (FORC, a measure of magnetic interaction Hu versus microcoercivity Hc) showing large interactions (correspondingly large spread of Hu values; compare with ideal single domain case (Supplementary Fig. 1b) where Hu is restricted and the central FORC peak is more than order of magnitude greater than that seen in Allende data). For FORC parameters, see Supplementary Fig. 2. **c** Magnetic susceptibility versus temperature in an Ar atmosphere. VT, Verwey transition; mag, magnetite. **d** Magnetic susceptibility cycling at successively higher temperatures measured in an Ar atmosphere. Red: heating, Blue: cooling. **e** Partial thermal remanent magnetization (pTRM) between 292 °C and room temperature (i, iii, v, vii) followed by demagnetization (ii, iv, vi, viii). All experiments conducted in an Ar atmosphere. Red horizontal line: predicted values. **f** Orthogonal vector plot of demagnetization of remanence acquired after cooling from 340 °C in a reversing applied magnetic field (polarity changes illustrated by bar chart). Field applied along z axis; Red: inclination. Blue: declination. Experiments in Ar; temperature labels are ^∘^C. Interval of medium temperature component (MT) interpreted in prior work as holding a primary remanence highlighted. **g** Backscatter scanning electron microscope image; red box show enlargement area (**h**). Arrows highlight exsolution textures. **h** Energy dispersive spectroscopy analyses: i. Fe-sulfide, ii. Fe-Ni sulfide and iii. Ni-Fe metal.

Paleointensity estimates for Allende reported using varied methods over different ranges of unblocking temperature and coercivity (Supplementary Table S1) span 3 orders of magnitude (<1 to 1600 μT). But to be reliable absolute paleointensity recorders, defined here as obeying Thellier’s laws^8^ where thermoremanent magnetization theory is applicable, magnetic grains must exhibit single domain or single domain-like^18^ behavior, and they must be non-interacting. Magnetic hysteresis first order reversal curve (FORC) data collected on Allende samples dominated by matrix (see Methods) are strikingly different from those meeting these requirements (Fig. 1a, b and Supplementary Figs. 1–3). Allende FORC data are not dominated by single domain grains but instead show pseudo-single to multidomain behavior with an unusually high coercivity (see Supplementary Information Section 1). While the presence of multidomain grains is a concern, a larger issue regarding recording fidelity is posed by the magnetic interactions seen in the data, which are much stronger than ideal magnetic recorders. However, given the multi-phase magnetic mineralogy of Allende, it is not possible from the magnetic hysteresis data alone to unambiguously identify the source of the interactions and thus its effect on remanence.

Prior studies have concluded that Allende’s remanence is carried by an Fe-sulfide; some assert a monoclinic pyrrhotite phase^10^. However, Allende’s low field magnetic susceptibility (Methods, Fig. 1c, Supplementary Information Section 1) is dominated by Fe-Ni phases (predominantly awaruite, Ni_3_Fe) and magnetite (Fe_3_O_4_), the latter showing evidence for the Verwey transition. This large difference between grains carrying remanence and magnetic susceptibility is unusual in terrestrial rocks known to be reliable paleointensity recorders. When cycled from low to successively higher temperatures, through the temperature range carrying the remanence, magnetic susceptibility data define the growth of an anomaly between ~200 and 270 °C (Fig. 1d), during which Allende increases its capacity to record an applied magnetic field. On cooling, a portion of this increased capacity is retained. These changes are reminiscent of the lambda transition in hexagonal pyrrhotite: weakly magnetic antiferromagnetic pyrrhotite changes to a strongly magnetic ferrimagnetic state through vacancy reordering^8^.

To explore directly the apparent unblocking temperature interval carrying magnetic remanence, a partial thermoremanent magnetization (pTRM) was imparted, thermally demagnetized, and re-imparted in cycles (Fig. 1e; Supplementary Information Section 2). Contrary to predictions, subsequent pTRMs result in higher intensities. Repeatable near zero values after demagnetization argue against laboratory thermal alteration (although this is sub-sample dependent; Supplementary Fig. 4). Instead, this behavior appears to be the remanence equivalent of magnetic susceptibility cycling (Fig. 1d) results where the material increases systematically its magnetization capability. We also observe non-systematic remanence increases or decreases (Supplementary Fig. 4) in zero field heating of pTRMs applied at higher temperatures (620 to 490 ^∘^C), also contrary to predictions but consistent with strong magnetic interactions.

Allende remanence data shows evidence for multiple components of magnetization (Supplementary Fig. 5), which fall mainly in one quadrant. A fusion crust test that would exclude terrestrial contamination of Allende data^10^ is ambiguous because low unblocking temperature magnetizations (natural remanent magnetization to 190 ^∘^C) within millimeters of the crust are indistinguishable from the key interior magnetizations (Supplementary Fig. 5, Supplementary Information Section 3). While composed of multiple components, it has nevertheless been proposed that the remanence is “quasi-single” component in nature and that it was acquired over millions of years, incompatible with a time-varying external source^10^. This interpretation assumes that magnetic unblocking during laboratory demagnetization is equivalent to magnetic acquisition (blocking) in nature. To test this equivalence, we heated Allende samples and changed the polarity of an applied field during cooling (see Methods, Supplementary Fig. 6, Supplementary Information Section 4). This treatment reproduces the “quasi-single” component magnetization (Fig. 1f). In contrast, experiments on terrestrial basalt samples differ, revealing imparted reversals (Supplementary Fig. 7, Supplementary Information Section 4). These experiments indicate that Allende magnetic carriers lock in the field to which they are first exposed during cooling and positive interactions during subsequent cooling result in a “quasi-single” component remanence. The lack of evidence for acquisition over long time intervals suggests that impacts^19,20^ remain a potential source for Allende’s magnetization.

Energy dispersive X-ray spectroscopy and electron microprobe data (see Methods, Supplementary Information Section 5), show that FeNi-metal grains commonly occur in close association with FeNi-sulfides and Fe-sulfides (Fig. 1g, h, Supplementary Fig. 8), providing mineralogical context for magnetic interactions. We interpret fine textures as reflecting complex exsolution between low Ni pentlandite^21^ and pyrrhotite (Fig. 1g, h). Exchange interaction between monoclinic and hexagonal pyrrhotite phases formed during exsolution may explain Allende’s rock magnetic behavior, including the apparent high coercivity seen in the FORC analyses (Supplementary Fig. 8, Supplementary Information Section 6). Regardless of the exact interaction mechanism, the very evidence for profound interactions, as well as the evidence for reordering indicates that Allende lacks paleointensity recording requirements and that the prior nominal value^10^ of ~20 μT, as well as other numbers (Table S1) have no basis in paleointensity estimation otherwise solidly founded on the physics of the magnetization process^8^. Importantly, this also extends to non-thermal mechanisms for estimating paleointensity which arises from a TRM in Allende (see Supplementary Information Section 6). Instead, previous values are physical property measurements of intrinsic magnetic interactions. Thus, we conclude that Allende’s magnetism is compatible with an undifferentiated parent body^11,16^, but that it cannot otherwise be used to shed light on ambient fields in the early Solar System.

Paleointensity analyses of the CV chondrite Kaba (CV3.1), however, are viable given the single domain-like (i.e., pseudo-single domain) magnetic carriers of magnetite composition^22^, without the extreme interaction and reordering problems when the dominant remanence carrier is a multiphase pyrrhotite (Supplementary Information Section 7). Kaba samples are dominated by viscous remanent magnetizations (VRMs)^22^, but the analysis that appears least affected by VRM yields a field of ~0.4–1.1 *μ*T. This is similar to that obtained from CM carbonaceous chondrites which have remanences carried by magnetite (i.e., Murchison, paleointensity estimate of approximately ~1 μT derived in two independent studies^23,24^, see Supplementary Information Section 7). We explore below whether external fields can explain these values.

CV asteroid metasomatism occurred at ~4562 million years ago (Ma)^5^, ${\sim} 4.{2}_{-0.7}^{+0.8}$ m.y. after CV CAIs and textural evidence indicates magnetite formation was relatively rapid (Supplementary Information Section 7). This is ~1.5 m.y. after cooling of volcanic angrites from the inner disk isotopic reservoir in a field  <0.6 μT^25^ interpreted to record magnetization after nebular dispersal which otherwise might be a magnetization source^26^. Solar wind ram pressure was much higher at these times due to the initial faster spin of the Sun, which in turn is associated with greater magnetic activity and higher rates of mass loss^27^. If the gyro-radius of the stellar wind ions is small compared to the asteroid, field can pile up given obstacles to slow the solar wind, expected for early solar system conditions (Supplementary Information Section 8). The ion gyro-radius scales as ${r}_{g} \sim 250\;{\rm{km}}{({B}_{0,1{\rm{AU}}}/4nT)}^{-1}$ where *B*_0,1AU_ is the field at 1 AU. This condition is not met for the present day solar wind. For solar-type stars 100 times younger, however, the product of surface field and rotation rate can be >20 times larger^28,29^, increasing the toroidal field at 1 AU by this factor in a Parker spiral model, and reducing *r*_*g*_ to <12.5 km. Correspondingly, the ratio of the Bohm diffusion time to wind crossing time for an asteroid of radius *r*_*b*_ ~ 20*r*_*g*_ satisfies ${t}_{dif}/{t}_{w}\ge {(20{r}_{g})}^{2}/({c}_{s}{r}_{g})({v}_{w}/20{r}_{g}) \sim 20({v}_{w}/{c}_{s}) \sim 100$ assuming a wind sound speed *c*_*s*_ = 100 km s^−1^, and wind velocity *v*_*w*_ = 500 km s^−1^. Under these circumstances, the flow may advect and drape the field around the asteroid.

As long as the magnetic diffusivity of the body *ν*_*b*_ is small enough, the field can accumulate near the surface until the magnetic force density (the sum of magnetic pressure and tension) balances that from the incoming wind ram pressure^30^. The amplification factor, expressed as the ratio of the maximum amplified field strength in the body *B*_0_ to the incoming wind field strength *B*_*i*_ that we expect and confirm by our simulations is $A\equiv {B}_{0}/{B}_{i}\simeq (1+{\chi }_{v})\,\max \{1,\min [M\sqrt{\gamma \beta },{R}_{m,b}]\}\simeq M\sqrt{\gamma \beta }={(8\pi \rho )}^{1/2}{v}_{w}/{B}_{i}$. Here *ρ* is the wind mass density, *γ* is the constant pressure to constant volume heat capacity ratio (*γ* = 5/3, for adiabatic and *γ* = 1 isothermal) and the last two relations follow when the volume magnetic susceptibility *χ*_*v*_ ~ 0 and the magnetic Reynolds number associated with the body satisfies ${R}_{m,b}\equiv {v}_{w}{r}_{b}/{\nu }_{b}\ > \ M \sqrt{\gamma \beta }\ > \ 1$, where *M* ≡ *v*_*w*_/*c*_*s*_ is the wind Mach number and *β* is the ratio of wind thermal pressure to wind magnetic pressure. The above expression for *A* can be immediately simplified into an expression for the amplified field

1 $${B}_{0} =	\,{v}_{w}(1+{\chi }_{v}){(8\pi \rho )}^{1/2}\\ =	\,2.1\ \mu {\rm{T}}(1+{\chi }_{v})({v}_{w}/1000\ {\rm{km}}\ {{\rm{s}}}^{-1}){(n/1000\,\,{{\rm{cm}}}^{-3})}^{1/2},$$

where we have scaled the wind speed and number density *n* (assuming a hydrogen dominated wind) to values possible for an early solar wind (1–5 Myr-old) for which the average x-ray luminosity, mass loss rates, and ram pressure could be 10^4^ times larger^30,31^ than for the present Sun. A giant stellar flare could also transiently increase the wind ram pressure by another factor of ~10, increasing *B*_0_ by another factor of 3.

**Fig. 2:**
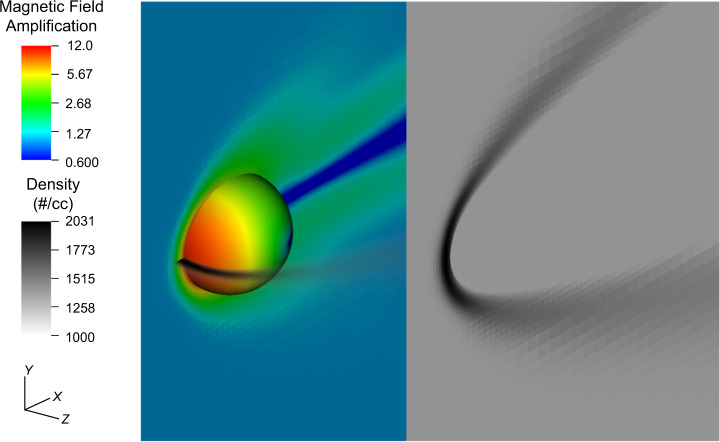
Magnetohydrodynamic numerical simulation results. Magnetic field amplification (color in logscale) along xy midplane and immediately above surface of asteroid (see Methods). Grayscale shows the number density (linear scale) in xz midplane as well as along xy midplane (transposed for visibility).

Using ideal 3D magnetohydrodynamic (MHD) simulations (Fig. 2, Methods, Supplementary Information Section 8, and Movie S1) for *χ*_*v*_ = 0 we observe high amplification factors $A\approx 12.25 \sim 1.07M\sqrt{\gamma \beta }$, confirming the scaling relations above. The latter similarity implies that equation (1), which depends only on the assumed wind speed and wind particle density and was derived from an amplification factor of exactly $M\sqrt{\gamma \beta }$, is a good estimate for the field amplified at the surface of the asteroid predicted by the simulations.

**Fig. 3:**
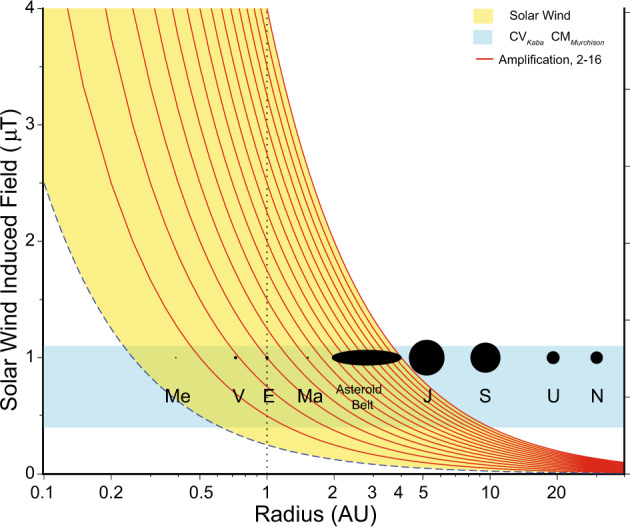
Solar wind interactions with young solar system asteroids. Solar wind induced field versus radius of orbit. Dashed line: minimum field limit (0.25 μT at 1 AU). Red lines: field amplified by factors (A) between 2 and 16. Yellow region: range of fields capable of magnetizing CV and CM chondrites as magnetic minerals either cool through their Curie temperature or grow through a blocking volume during metasomatism. Blue: CV_Kaba_ and CM_Murchison_ are select paleointensity values from the Kaba and Murchison meteorites (see text and Supplementary Information Section 7). Present-day location of planets and asteroid belt shown diagrammatically for reference: Me, Mercury; V, Venus; E, Earth; Ma, Mars; J, Jupiter; S, Saturn; U, Uranus; N, Neptune.

The CV magnetization constraints (both field strength and directions, see Supplementary Information Section 8) can be met at orbital distances consistent with the current asteroid belt (~2–4 AU) by these amplified solar wind induced fields, or by smaller amplifications and orbital distances (we view the former as more likely) (Fig. 3), representing the first direct tracking of the heliocentric distance of a meteorite parent body using paleomagnetism^32,33^. Aqueous alteration on the CM parent body occurred at ~4.8 m.y. after CAI formation^4^, essentially contemporaneous with CV metasomatism. CM magnetizations are also satisfied at ~2–4 AU, but not at much greater distances.

The arrival of the CV and CM parent bodies at or within heliocentric distances of the asteroid belt from an outer disk isotopic reservoir by the magnetization age of ca. 4562 Ma places further constraints on early Solar System events. Prior to disk dispersal, the accretion stream onto the Sun would have shielded asteroids from the solar wind, possibly resulting in even smaller field values. Accretion rates drop rapidly after giant planets have opened >~1 AU-scale gaps (transitional disks), and the Solar System must have gone through this phase shortly after the formation of Jupiter and Saturn. Once accretion rates fall below ~10^−10^ Solar mass yr^−1^, dissipation of gas and dust by photoevaporation, aided by stellar winds, is very rapid^34^. CV and CM external solar wind magnetizations thus indicate that accretion rates had fallen below this threshold, and that gas and dust were essentially dissipated, before 4562 Ma.

## Methods

Experiments were conducted at the University of Rochester Paleomagnetic Laboratories (UR) and the Michigan Technological University Earth Magnetism Laboratory (MTU). Three separate Allende samples were studied; two were obtained commercially and one from a university collection. Subsamples analyzed were at least 1 cm from the fusion crust. Magnetic susceptibility data were collected at both the UR and MTU labs using an AGICO KLY-4S Spinner Kappabridge equipped with a CS-3 Furnace Apparatus and a CS-L Cryostat and an AGICO MFK1-FA Kappabridge equipped with a CS-3 Furnace Apparatus and a CS-L Cryostat, respectively. Magnetic hysteresis data, including FORC analyses^35^ and were collected using a Princeton Measurement Corporation Model 2900 Alternating Gradient Force Magnetometers. For all magnetic hysteresis and remanence measurements 1-2 mm-sized un-oriented subsamples were selected to target matrix material and exclude large visible chondrules, (minimizing the effects of inverse TRM recently reported from a study of Allende chondrules^36^). A 3 component 2G DC SQUID magnetometer with high resolution sensing pickup coils access bore 4.2 cm, sensitivity ~5 × 10^−12^ A m^2^) in the UR magnetically shielded room (ambient field  < 200 nT) was used for remanence measurements. Thermal demagnetization and pTRM acquisition experiments were conducted using a CO_2_ laser^37–40^ in conjunction with a new controlled atmosphere chamber (e.g. Argon or Nitrogen) and additional magnetic shielding to produce a magnetically null environment (for field-off demagnetization) or an applied field coil (for field-on experiments). The non-magnetic atmosphere chamber is outfitted with a ZnSe window that permits transmission of the CO_2_ laser beam, while maintaining the gas atmosphere within the chamber during thermal experiments. Allende samples were mounted on fused quartz rods with sodium silicate and minor amounts of Omega cement.

Low to intermediate temperature (0–292 °C) pTRM experiments involved cycles, in either air, argon or nitrogen of the application of a pTRM at 292 °C in a 30 μT field, followed by a heating to 292 °C in the absence of a field. In high to intermediate temperatures (620–490 °C) pTRM experiments, a pTRM was imparted by heating to 620 °C in the absence of a field and cooling in a 30 μT field to 490 °C, after which the applied field was turned off and the sample was cooled to room temperature. The sample was subsequently heated to 292 °C, 310 °C, and 490 °C in zero field. An infrared pyrometer (FLIR ThermaCam SC640) was used to measure cooling curves for Allende samples and a terrestrial basalt. The basalt sample is a clast in a hyaloclastite (Ocean Drilling Program Leg 197, Hole 1206A-7R-1, Int. 128–130 cm)^41,42^. Calibration samples were heated with a CO_2_ laser outside of the controlled atmosphere chamber and cooled with Ar flowing across the face of the sample to simulate flow of argon during the experiments. Allende and basalt samples were heated to initial temperatures of 340 °C and 260 °C, respectively, to ensure that enough time was spanned during cooling to apply at least one field reversal within the remanence carrier unblocking range. A switch was constructed to allow an instant transition between field polarities. The applied field strength for all experiments was 60 μT and all heatings were in Ar. To limit potential alteration, Allende samples were not demagnetized prior to imparting the pTRM; the peak pTRM temperature of 340 °C however ensures that dominant remanence has been demagnetized. Basalt samples were demagnetized prior to the pTRM step to evaluate unblocking temperature range of remanence carriers for the samples.

Scanning electron microscopy (SEM) and energy dispersive spectroscopy (EDS) analyses were conducted using a Zeiss Auriga Scanning Electron Microscope with an EDAX spectrometer at the University of Rochester Integrated Nanosystems Center. Samples were prepared in an acrylic mount and given a final polished surface using colloidal silica prior to mounting and carbon coating for SEM analyses. Additional analyses targeting the Fe-Ni-S system were made using a Cameca SX-100 electron microprobe at Rensselaer Polytechnic Institute’s Electron Microprobe Laboratory. The microprobe was calibrated to measure S, Fe, O, Ni, and Si using the following respective standards: FeS_2_ (2 standards), magnetite (Fe_3_O_4_), nickel, and kyanite. Data were collected with an accelerating voltage of 15 keV and 10 keV.

We investigated a magnetized solar wind overrunning an asteroid with the AstroBEAR^43^ adaptive mesh-refinement code. The young stellar wind was given a density of 1000 particles cc^−1^, a temperature of 10^6^ K, and was traveling at 500 km s^−1^ (in *x*) carrying a perpendicular magnetic field of 100 nT (in the *y* direction) into a magnetized ambient at the same density and temperature. The stellar wind had a Mach number of 4.74, a beta of 3.47, and an Alfvenic Mach number of 8. We used the equations of resistive MHD with a specific heat ratio of 1.667 to model the wind as a monoatomic gas. The asteroid was modeled as a 500 km radius solid boundary centered at the origin, which does not act as a significant source of particles for flows diverging from the surface. The asteroid has a conductive outer layer, i.e., its 10% outer shell on the day side is 10 times more conductive than the parent body, to simulate a cometary-like solar wind interaction (see Supplementary Information Section 8). Diffusion was turned on for all flows outside the asteroid to speed up convergence. The solution results can be rescaled to arbitrary asteroid radius provided the asteroid radius  >  gyration radius. The wind density, temperature, velocity, and magnetization can be rescaled provided the same magnetic beta and Mach number are used. The simulation boundaries are extrapolated and sufficiently far away, such that the magnetic field is perpendicular as it leaves the box. The base resolution was 64 × 64 × 64 with 4 additional levels of adaptive mesh refinement (AMR) around the asteroid allowing us to resolve the asteroid’s diameter with 128 zones.

## Supplementary information

Description of Additional Supplementary Files

Supplementary Information

Supplementary Movie 1

## Data Availability

Data are available on the Earthref.org MagIC database (earthref.org/MagIC/16980).
