# Supplementary material for: Arrival and magnetization of carbonaceous chondrites in the asteroid belt before 4562 million years ago
Source: Commun Earth Environ. 2020 Dec 4;1(1):54. doi: 10.1038/s43247-020-00055-w (PMC7716897; doi:10.1038/s43247-020-00055-w)
Supplement: Supplementary file 1 — Description of Additional Supplementary Files [file 43247_2020_55_MOESM1_ESM.pdf]

Description of Additional Supplementary Files

File Name: Supplementary Movie 1
Description: Select magnetic field lines drape over the surface of the asteroid (shown in transparent gray). The field lines are colored by their strength relative to the incoming solar wind values. The field lines were integrated from points evenly spaced along a line across the bottom boundary (y = âˆ’8r_asteroid & z = 0), as well as from points within a circle of radius 3r_asteroid centered at the center of the asteroid. The slice inside the asteroid shows the azimuthally symmetric resistivity structure. The asteroid is stationary to highlight the field amplification. To see how this relates to magnetization acquisition for a rotating asteroid, see Parent body rotation in the Supplementary Information document.
