# Supplementary material for: Arrival and magnetization of carbonaceous chondrites in the asteroid belt before 4562 million years ago
Source: Commun Earth Environ. 2020 Dec 4;1(1):54. doi: 10.1038/s43247-020-00055-w (PMC7716897; doi:10.1038/s43247-020-00055-w)
Supplement: Supplementary file 2 — Supplementary Information [file 43247_2020_55_MOESM2_ESM.pdf]

October 6 2020

Supplementary Materials for "Arrival and magnetization of carbonaceous chondrites in the asteroid belt earlier than 4562 million years ago"

Timothy O'Brien, John A. Tarduno*, Atma Anand, Aleksey V. Smirnov, Eric G. Blackman, Jonathan Carroll-Nellenback, Alexander N. Krot

*To whom correspondence should be addressed. E-mail: john.tarduno@rochester.edu

Supplementary Section 1

Magnetic Hysteresis Data

A Day plot of Allende magnetic hysteresis values shows nominal pseudo-single domain (PSD) to multi-domain (MD) remanence carriers (Supplementary Figure 1a) consistent with previous work[1-4]. We note such identifications (using a Day plot) are nominal because of the complex multiphase magnetic mineralogy of Allende. First Order Reversal Curve (FORC) data (Supplementary Figure 1, d and f) must also be interpreted with care because of the presence of multiple magnetic phases. Nevertheless, the FORC data also indicate PSD to MD carriers. In particular, these data are very different from those collected on samples with ideal single domain grains (Supplementary Figure 1b) and instead display a wide spread of Hu values indicating the presence of magnetic interactions. Expanded FORC plots are shown in Supplementary Figure 2 for reference. Additional processing is shown in Supplementary Figure 3. The lower branch was subtracted from all FORCs before smoothing. The following smoothing parameters were used: Sc0 = 4, Sb0 = 3; Sc1, Sb1 = 7; horizontal and vertical $\lambda = 0.3$. Further FORC analyses could include use of a finer field increment, but in any interpretation one must be mindful of potential non-uniqueness when multiple magnetic phases are present. Instead, we prefer to focus on the remanence carrying magnetic phases, as discussed below.

Magnetic Susceptibility Data

Bulk magnetic susceptibility data show evidence for the Verwey transition[5], where magnetite transforms from cubic (temperatures $>$ -153 ^{o}C) to monoclinic (temperatures $<$ -153 ^{o}C) symmetry. Macke et al.[6] summarize prior bulk susceptibility measurements on Allende, expressing mass normalized values as log χ in log units of 10^{-9} m^3 kg^{-1}. Our mass-normalized data (Fig. 1C) yield log χ=3.75, within the range (3.43-3.84) reported in prior studies. The growth of magnetic susceptibility with multiple heating/cooling sequences (Fig. 1D) is similar to the λ-transition of hexagonal pyrrhotite, whereby antiferromagnetic undergoes a thermally induced transition to a ferrimagnetic structure[7-8]. The new magnetic state is quenched by cooling rates $>$ 0.5 ^{o}C min^{-1}; a cooling rate value typically exceeded in our thermal experiments. We interpret changes in the temperature of the peak manifestation of the λ-transition to denote progressive vacancy ordering. Finally, the changes we note have similarities to data reported by Wasilewski and Saralker[9] in their study of Allende chondrules. In that study the magnetization was also inferred to be carried by pyrrhotite and interactions were hypothesized.

Supplementary Section 2

Remanence Measurements

The maximum temperature value of $\sim$292 ^{o}C for pTRM experiments examining the dominant remanence held by Allende was chosen on the basis of prior works[10]. The null hypothesis for these experiments is that successive applications of a pTRM at the same temperature will not result in a change in magnetic intensity

(relative to the pTRM). The null hypothesis is represented by a horizontal line labeled "Expected" on each plot on Supplementary Figure 4. None of the subsamples shows behavior compatible with the null hypothesis. Potential processes accounting for the data include magnetic interactions, order-reordering, creation of a new magnetic phase, and magnetic mineral destruction (conversion to a non-magnetic state). The role of these processes is likely sub-sample dependent. For example, Supplementary Figure 4a (conducted in Nitrogen) demonstrates a steady increase in the pTRM remanence, but also suggests a small magnetic component that is not fully demagnetized (step 2). This component decreases with subsequent steps and therefore appears to be decoupled from the trend of growing pTRMs. In Supplementary Figure 4b (specimen heated in Argon), a more irregular pattern is seen, which affects both the pTRM growth and the undemagnetized phase. In the specimen shown in Supplementary Figure 4c (heated in Argon), the un-demagnetized phase is very minor but does grow slightly with the number of heatings. In the specimen shown in Supplementary Figure 4d (conducted in Argon), the remanence after demagnetization is negligible, whereas for the specimen shown in Supplementary Figure 4e (conducted in Nitrogen), a large increase in the undemagnetized component in seen with sequential heatings. Interestingly, one specimen measured in air (Supplementary Figure 4f), showed a pattern nearly identical to one measured in Argon (Fig. 1e, main text), with complete demagnetization and growth of pTRMs. Irrespective of the sub-sample variations, the salient trends are best reflected by the samples which show complete (or nearly complete) demagnetization of the pTRM and steady growth of the subsequent pTRM (Fig. 1e main text; Supplementary Figure 4d, 4f). Samples subjected to a series of heating/cooling steps (0-292 ^oC, 0-340 ^oC, 0-490 ^oC) in the absence of a field after the application of a pTRM at higher temperatures (between 620 and 490 ^oC) should also not show directional or intensity change. Instead (Supplementary Figure 4g-i), there is a clear deviation of the directional and intensity values from the expected results and the null hypothesis can be rejected. This deviation may be the result of magnetic interactions and/or reordering, and again the changes appear to be subsample dependent.

Supplementary Section 3

Allende Fusion Crust

Prior works[10−11] have sought to evaluate the impact of fusion crust formation and associated heating in Earth's atmosphere and magnetic field on the interior Allende material. In ref 11, NRM directions for these materials were compared (Supplementary Figure 5a). A trend of magnetizations from the interior material to the magnetization of the fusion crust was found. This trend appeared to support long held inferences, based on a consideration of the low thermal conductivity of chondritic meteorite, that only the outer millimeters of Allende material would be significantly heated by passage through the atmosphere. However, there are important caveats on this conclusion. In ref 11 only the undemagnetized natural remanent magnetization (NRM) was compared. In addition, the mineralogy and grain size (and associated domain state) of magnetic carriers in the fusion crust likely differ from that of the interior material. The undemagnetized NRM is an inconclusive value for assessing the nature of potential primary meteorite magnetizations because it contains secondary magnetizations (at least including viscous remanent magnetizations, VRM). Hence, *sensu stricto*, the results of ref 11 only document that the undemagnetized fusion crust magnetization differs from the VRM-dominated interior magnetization, and not that the partially demagnetized interior of Allende is unaffected by the influences of passage through the atmosphere.

Demagnetized NRM directions were presented in ref 10, but these raise further questions (Supplementary Figure 5b). These authors compared the low (LT), middle (MT), and high (HT) temperature directional components of interior and fusion crust samples. The LT component of the fusion crust is essentially indistinguishable (data and 95% uncertainly regions overlap) from the critical MT component of the interior on which the authors base their conclusions on an ancient magnetization for Allende. The LT component of the fusion crust is argued to represent a contamination of unmelted (and hence un-remagnetized) material from the interior. In support of this interpretation, the authors note that the NRM of their 2 samples containing the largest fraction of fusion crust have the lowest contribution of "LT+MT" components. But this does not comment on the fundamental conundrum. That is, the mixing model of ref 10 focuses on the HT fusion crust component. The fundamental ambiguity, however, is the agreement of the LT fusion crust magnetizations and the interior MT component, so the focus should be on separating the LT fusion crust magnetization. This component is isolated at very low temperatures (0-190 ^oC) from mm-sized specimens

that are reported to be adjacent to the melted fusion crust. Regardless of low thermal conductivity, it is highly unlikely that unmelted material adjacent to melted material on a millimeter scale would not be heated to at least a few tens of degrees C (note, meteorite material > several mm from this zone might well remain pristine). Importantly, we should not expect the HT and LT components from the fusion crust to agree if both were acquired during passage through the atmosphere and subsequent cooling: the HT component represents the quench of a melt at high temperature whereas the LT fusion crust component would represent a longer cooling process (even if on timescales of seconds). The meteorite is unlikely to have maintained a constant orientation, and thus the expectation is that the LT and HT components should differ. As predicted, the fusion crust LT and HT components are different in the data of ref 10. But the former is again indistinguishable from the key MT component instead of representing some third direction. In summary: the LT component of the fusion crust isolated at 0-190 ^{o}C in ref 10 should bear a component different from the interior magnetization; it does not and this represents a fundamental paradox. Ultimately, the inability to completely isolate the fusion crust material[10], and the unresolved similarity of the LT fusion crust and interior sample magnetizations results in an ambiguous outcome for the fusion crust test.

Evidence for multicomponent magnetizations

Some prior studies[10,12] report a "uniform" directional middle-temperature (MT) component unblocked between 190-290 ^{o}C and 100-$\sim$280 ^{o}C respectively. In ref 10 the low-temperature (LT) component (0-190 ^{o}C) is attributed to a viscous remanent magnetization (VRM). The claimed uniformity of the MT direction has been interpreted to be strong evidence for a parent body internal magnetic field for Allende. However, in earlier work on Allende[13], at least 3 to 4 components of magnetization were identified in vector plots of thermal demagnetization (Supplementary Figure 5, c and d), although it should be noted a limited number of demagnetization steps were applied. As is standard practice, components are identified by breaks in slope seen in orthogonal vector plots. As compared to the interpretation of the earlier work[13], the vector plots of ref 10 are similar, suggesting the presence of up to four components of remanence (Supplementary Figure 5, e and f). Importantly, one thermal experiment reported in ref 10 yielded negative inclinations for the MT component, compared with positive inclinations for other samples (Supplementary Figure 5g). Thus, we conclude that a single uniform MT component is not unambiguously supported by the data presented in ref 10, or that of other studies. Instead, the available data are suggestive of multiple remanence directions which may be poorly resolved/recorded because of extreme magnetic interactions.

Supplementary Section 4

Cooling in a Reversing Field (CIRF) experiments

According to Thellier's laws[7] whereby magnetizations blocked in sequential temperature increments are independent (and thus the sample is capable of recording changes on the inducing magnetization with time). To test whether Allende has the requiring recording fidelity, a series of CIRF experiments were performed. Calibration values are likely upper bounds on sample cooling rates because calibration samples were laser heated outside of the controlled atmosphere chamber and cooled with Ar flowing across the face of the sample (this was necessary to allow access of the IR pyrometer). While the long-term sample cooling behavior appears to be convective (exponential), the first few seconds of cooling may be dominated by radiative heat transfer[14]. For Allende, experiments were conducted with polarity reversals every 5 seconds (Supplementary Figure 6) and with polarity changes every 3 seconds for the first 30 seconds, followed by 5 second switching intervals (Fig. 1f, main text). The 3 and 5 second switching times were decided upon by balancing the need to capture several reversals within the unblocking temperature range examined and time needed to change the field (in the experimental setup utilized).

Allende 5 sec reversal experiment results

The pTRM was applied in a positive or negative z direction (90° or -90° inclination). In the 5 sec reversal experiments, Allende samples maintained a quasi-linear component of unblocking through the MT component range. Variability was observed between subsamples in the linearity of the directions. For most samples, the demagnetization data define a nearly linear trajectory (Supplementary Figure 6, b-d) whereas in one

(Supplementary Figure 6e) there is curvature. For some samples (Supplementary Figure 6, c and d) there is an apparent reversal at low unblocking temperatures, below 200 ^{o}C.

Terrestrial basalt reversal experiment results

For the examination of a terrestrial sample for our CIRF analyses, we sought a specimen whose remanent magnetization was dominantly observed at low unblocking temperatures. We selected a sample of a typical Hawaiian-Emperor basalt obtained by ocean drilling[15−16]. Basalts from the Hawaiian-Emperor chain are typically dominated by titanomagnetite carriers with varying amounts of Ti and low temperature oxidation. Basalt specimens were demagnetized in Ar prior to conducting a reversal experiment to confirm the expected low dominant unblocking temperatures consistent with a titanomagnetite carrier[15−16] (Supplementary Figure 7, a and b). We do note, however, that specimens vary in their maximum unblocking temperature, most likely reflecting subtle differences in titanium content. Chips from the basalt sample examined yielded single-domain hysteresis curves (Supplementary Figure 7c). After heating to 260 ^{o}C, samples were cooled in an applied field with reversals every 5 seconds (Supplementary Figure 7, d-f). An additional experiment was conducted with field reversal intervals of 3 seconds for the first 30 seconds followed by 5 second intervals (Supplementary Figure 7, g and h). Thus, these reversal durations duplicate those applied to the Allende samples. For the 5 second data, reversals are captured, but there is some sample variability. For sample 06AR71_a, there is a clear field reversal in the dominant unblocking temperature range. For sample 06AR71_c, a reversal is seen at somewhat lower unblocking temperatures. The lack of multiple reversals in these samples may indicate differences between blocking and unblocking characteristics, limited magnetic grains with blocking/unblocking characteristics in some temperature intervals. The 3 sec/5 sec experiment also shows a reversal in the dominant unblocking temperature range, but not multiple reversals in the lowest unblocking interval where only small amounts of remanence are held. Overall, the presence of reversals these experiments is in stark contrast to the data from Allende.

On the basis of these results, we further propose CIRF experiments as a general protocol for evaluating Thellier's laws[7] in meteorites, and hence their suitability for preserving paleofields. While meteorites are susceptible to thermally-induced alteration, we emphasize that only thermal methods can truly evaluate their TRM recording fidelity.

Supplementary Section 5

SEM and Microprobe analyses of sulfide particles

SEM was used to target potential magnetic remanence carriers within Allende (e.g. Fe-Ni sulfides, iron sulfides, oxides, and iron-nickel alloys), with a particular focus on matrix material. The common association of Fe-Ni sulfides with awaruite (Supplementary Figure 8a), noted by Clarke et al.[17], suggest the growth of a secondary Fe-S and Fe-Ni-S phases at the expense of awaruite. SEM analyses of these relationships in Allende suggest a system in which the progressive metasomatism on the CV parent body resulted in alteration of preexisting kamacite to awaruite and Fe-Ni sulfide phases including pentlandite and potentially pyrrhotite (see Fig. 1h, main text, analysis "1"). The pyrrhotite phase represents a small fraction of the Fe-Ni sulfide grains and is commonly observed as possible exsolution features (Fig. 1g) or separate phases within Fe-Ni-S (Supplementary Figure 8b). Additional geochemical work was performed to target the Fe-Ni-S system within Allende using a Cameca SX-100 electron microprobe at Rensselaer Polytechnic Institute's Electron Microprobe Laboratory. The microprobe was calibrated to measure S, Fe, O, Ni, and Si using the following respective standards: FeS_2 (2 standards), magnetite (Fe_3O_4), nickel, and kyanite. Data were collected at an accelerating voltage of 15 keV. Measurements were also collected at 10 keV targeting Fe-S and Fe-Ni-S phases at a higher spatial resolution in an attempt to distinguish a pyrrhotite or non-stoichiometric pentlandite phase from the non-magnetic troilite and pentlandite phases. These data, targeted Fe-Ni-S system grains throughout the Allende matrix (Supplementary Figure 8b), suggest four or five phases: awaruite, pentlandite, pyrrhotite/troilite, and a Fe-S phase with minor nickel. The phases of interest for magnetization are the low-Ni Fe-S phases. We note that the low-Ni non-stochiometric "pentlandite" (Supplementary Figure 8b), may also simply be a pyrrhotite phase with minor nickel in the lattice as has been observed by Vaughan et

al.[18] in terrestrial sulfides. The inclusion of nickel would explain the deviation from characteristic behaviors of typical terrestrial Fe-Sulfides (e.g. lower Curie temperature).

Supplementary Section 6

Model for Allende magnetic interactions

In prior works invoking a CV core dynamo Allende's magnetization is interpreted to be TRM. This linkage is key in using paleointensity values to constrain processes, because magnetization theory is only available for TRM, providing the foundation for the interpretation of absolute paleointensity values. Non-thermal paleointensity measurements have been applied to Allende to estimate TRM paleointensity (table S1). However, because Allende does not preserve a reliable TRM signal of ambient fields, non-thermal methods to estimate its "TRM" are similarly physical property measurements and not paleofield strength values.

While a detailed model is beyond the scope of this work, our preliminary model involves positive exchange interaction between monoclinic and hexagonal pyrrhotite found as exsolved phases within larger pentlandite grains. A hypothetical evolutionary history of the magnetic moments during cooling from temperature greater than the Curie temperature of pyrrhotite is shown in Supplementary Figure 8c. Because of the extreme interactions, the final moment does not relate to paleointensity following Thellier's laws or TRM theory.

Supplementary Section 7

Kaba CV3 Meteorite

The CV Kaba meteorite has opaque phases that represent $\sim$4% of the volume, consisting of magnetite, awaruite, pentlandite, and troilite[19–22]. While these consist of the same general assemblages observed in Allende, Kaba contains approximately 11 weight percent magnetite[23–24], highlighting its importance as a potential fidelity as a magnetic recorder. Thermal demagnetization data[23] on Kaba samples have been interpreted as being composed of a dominant low temperature (LT) viscous (VRM) component (NRM to approximately 180 ^{o}C) and a medium temperature (MT) component isolated between $\sim$180 ^{o}C and $\sim$250 ^{o}C (Supplementary Figure 9a). The interpreted MT component represents only $\sim$5% to 16% of the total NRM (e.g., Supplementary Figure 9c). Because incomplete removal of the VRM can result in erroneous paleointensity values, care must be taken to select data least affected by this effect. For example, two of the samples analyzed in ref 23 (Supplementary Figure 9, d and f) do not show a clear delineation between the LT VRM component and the MT component; these data do not demonstrate that the nominal MT component is not contaminated by VRM. In contrast, there is a clear directional difference between the the LT VRM and MT components for sample P1 (Supplementary Figure 9a), which yielded a paleointensity value of $\sim$1.1 $\pm$0.2 μT. This value is reduced to $\sim$0.6 μT after cooling-rate correction. Because Kaba's magnetization could represent a nearly instantaneous record (see "Magnetite formation in Kaba" below) this is probably an overcorrection. A "chondrule correction" addresses the assumption that, similar to assumed for Allende chondrules[12], the magnetization of the Kaba chondrules has been reset in a null field environment by late-stage metasomatism. It is not clear why such hypothetical fluids would not reset the matrix, nor is it clear how such chondrules would contribute to pTRMs in paleointensity experiments. Nevertheless, even if we consider a maximum effect, the paleointensity should only be corrected upward by a factor of 1.5, leading to a value of $\sim$0.89 μT for Kaba. Here we consider a conservative range, from the cooling rate corrected value (and its error), to the hypothetical chondrule corrected value (and its error), leading to 0.4 to 1.1 μT. We note that none of the Kaba orthogonal vector plots show MT components that trend towards the origin of orthogonal vector plots, indicating the presence of additional unresolved magnetic components at higher unblocking temperatures. This observation, together with the curved or irregular nature of the MT component (best seen in the relative declination of P1 and the relative inclination of L1) indicate that similar to Allende, the MT magnetization is itself composed of multiple components. The very small ($\sim$70 ^{o}C) unblocking temperature range where an approximately linear component is observed and where an approximate paleointensity estimate is available is compatible with a solar wind magnetization process (see Supplementary Section 8).

Magnetite formation in Kaba

For Kaba, the acquisition of magnetization was likely rapid, rather than on thousands to millions of years assumed in ref 23. Longer timescales may apply to the overall alteration on the CV parent body. But alteration was demonstrably heterogeneous, creating $CV3_{oxA}$ and $CV3_{oxB}$ types (and others). Kaba is a Bali-type (i.e., $CV3_{oxB}$), with heating temperatures <300 ^{o}C[25]. The relevant alteration is that related to the formation of magnetite. Heating most likely coincided with magnetite formation, so the magnetization is either a thermochemical remanent magnetization or a TRM. There are two major textural types of magnetite in Kaba. Magnetite pseudomorphically replaces Fe,Ni-metal nodules inside magnesian porphyritic (type I) chondrules. This magnetite contains high concentrations of minor elements (Cr, Ni, P) which were originally dissolved in Fe,Ni-metal and the inferred reaction is as follows:

$$4Fe_{(s)} + 4H_2O_{(liq,g)} \rightarrow Fe_3O_{4(s)} + Fe_{(aq)} + 4H_{2(g)}$$

Massive, compositionally pure magnetite is observed overgrowing the nodular magnetite. It is often connected to magnetite-bearing veins that crosscut fine-grained rims around type I chondrules. This magnetite often contains lath-shaped inclusions of Fe,Ni-sulfides. The inferred reaction for the formation of this magnetite is:

$$3Fe_{(aq)} + 4H_2O_{(liq,g)} \rightarrow Fe_3O_{4(s)} + 4H_{2(g)}$$

Both types of magnetite have identical O-isotope compositions, which are out of isotope equilibrium with chondrule olivine phenocrysts, suggesting late (after crystallization of chondrule melt) formation, in the presence of aqueous solutions. There is hence no evidence of O-isotope evolution of aqueous solutions and no evidence of multiple generations of magnetite. These observations, together with the relatively small magnetite grain size are incompatible with formation spanning thousands to millions of years and instead argue for a rapid formation event. The exact time scales are uncertain but magnetite can form from hydrothermal fluids on hour time scales[26]. Hence, the magnetite in Kaba over the relevant ~70 ^{o}C unblocking temperature range might record a nearly instantaneous ambient field on the CV parent body.

CM Chondrites

CM chondrites have both pyrrhotite and magnetite remanence carriers[27]. Here we focus of Murchison, which has a dominant magnetite carrier (see references 27 and 28 for a discussion of Murchison magnetic properties). Paleointensity estimates of ~1 μT have been reported from two groups[27−28]. We note that this magnetization must be mainly a chemical remanent magnetization because of the low temperature experienced on the Murchison parent body during magnetite formation. Because CRMs tends to underestimate field strength, this paleointensity value should be considered a minimum. Nevertheless, its agreement with Kaba values is notable.

Supplementary Section 8

Parent body/solar wind interaction

Our modeling applies for an object with a highly conductive boundary layer that stands off the solar wind. Carbonaceous chondrite meteorites contain hydrated silicates and thus water is expected to have been lost from their parent bodies early in solar system history. We follow Brownlee[29] who inferred that such bodies should be thought of as having cometary-like solar wind interactions. Thus, we assume that this is the dominant process creating a near-surface standoff of the solar wind. Accordingly, in our simulations we have incorporated a case where the day side of the 10% outer shell of the parent body is 10 times more conductive than the interior while the night side has the same conductivity, and another case where the entire shell is 10 times more conductive than the interior. Both cases yield similar amplifications in steady state for the parent body conductivity we used ($\sigma_b = 7.96 \times 10^{-4}\,\sigma m^{-1}$). We note that in a paper criticizing an abstract[30] report of our original hypothesis of solar wind induced meteorite magnetizations, Oran et al.[31] modeled the absorption of all incoming solar particles by the parent body; this is physically implausible.

Their modeling may be applicable to some aspects of current solar wind interactions with airless asteroids, but it is otherwise not relevant for hydrated early solar system parent bodies. Nevertheless, it is notable that Oran et al.[31] derived an amplification factor of the solar wind magnetization that is within a factor of 1.25 of our theoretical value. In their case, the solar wind pileup relates to low conductivity of the parent body. However, because they do not account for the expected cometary-like solar wind interaction, their modeling does not achieve the higher amplification values that accompany a balance of solar wind ram pressure with magnetic force density seen in our modeling. Further, their solar wind model with a density of 35 particles/cc, a temperature of $50,000$ K, and traveling at 700 km/s, has a much lower $M\sqrt{\gamma\beta} \simeq 3$, leading to a lower amplification of 3.6, as expected. They also base their conductivity value off the cooling curve of Sample 3 in Duba and Boland[32], which is lower than that of other meterorite samples. We ran a simulation with the same solar wind and resistivity parameters as Oran et al.[31] and obtained amplification values within 3%, which demonstrates that both code results are consistent with each other.

The extent to which the resistivity structure determines the magnetic amplification can be estimated crudely from the magnetic Reynolds number (R_m):

$$R_m \equiv \frac{L_{scale}\, U_{scale}}{\nu} = \frac{2r_b\, v_w}{\nu_b} \tag{1}$$

for a wind velocity of v_w, a parent body of radius r_b, and effective magnetic diffusivity ν_b. We define the equivalent electrical conductivity (σ_{eff}) as a weighted average w.r.t the current density

$$\sigma_{eff} = \frac{\int_{asteroid} \sigma \, |\nabla \times \mathbf{B}| \, dV}{\int_{asteroid} |\nabla \times \mathbf{B}| \, dV} \tag{2}$$

and note in SI units, $\nu_b = 1/(\mu_0 \sigma_{eff})$. Our case with a shell around the whole parent body has $R_{m,b} = 3413$, while the Oran et al.[31] case has $R_{m,b} = 794$. Our simulations suggest that all other factors being the same, cases with higher values of $R_{m,b}$ have higher amplifications in general, until they reach the saturation limit of $\approx M\sqrt{\gamma\beta}$.

Solar wind variability

Solar wind variability might at first glance appear to be incompatible with carbonaceous chondrite magnetic data. Below we provide further background why this is not the case. For the present-day Sun, an asteroid sampling the Parker spiral field will see a ∼25 day polarity reversal history[33]. Dynamics of the young Sun are uncertain[34] but it is likely that the magnetic and rotational axes were not aligned and that polarity reversals were at least as frequent as today. Thus, the variable polarity of the solar wind could impart different polarity magnetizations on a parent body, but understanding how this will ultimately be recorded by a meteorite sample depends on an in-depth study of rock magnetism as detailed above. For Allende, our CIRF experiments reveal that its magnetization is consistent with exposure to a reversing ambient field (of unknown intensity). The Kaba magnetic data also suggest multiple components that are not fully resolved. We also note that the unblocking temperature range over which a remanence (free of IRM effects) is isolated from Kaba is limited to a very small interval (∼70 ^{o}C), potentially consistent with a very short acquisition time suggested by textural relationships described below.

Parent body rotation

Fig. 2 also illustrates why a consistent magnetic direction can be imparted for a body undergoing a consistent rotation (note here we consider parent bodies >10 km in diameter that are immune to Yarkovsky-O'Keefe-Radzievskii-Paddack (YORP) effects that can induce tumbling[35]). The well-established thermoremanent magnetization theory dictates that the total observed vector is a linear combination of its component parts:

$$TRM = \sum pTRM \tag{3}$$

$$TRM = pTRM_1 + pTRM_2 + pTRM_3 + pTRM_4 + pTRM_5 \cdots + pTRM_n \tag{4}$$

where TRM is the total thermoremanent magnetization (resultant vector) and $pTRM_{1 \cdots n}$ are partial thermoremanent magnetization imparted over discrete temperatures during cooling. Given the sharp fall-off of field amplification (as illustrated in Fig. 2), the $pTRM$ terms near the magnetopause will dominate the TRM such that a quasi-linear component of magnetization is the expected result when conducting demagnetization experiments on meteorites from parent bodies magnetized by intense solar winds.

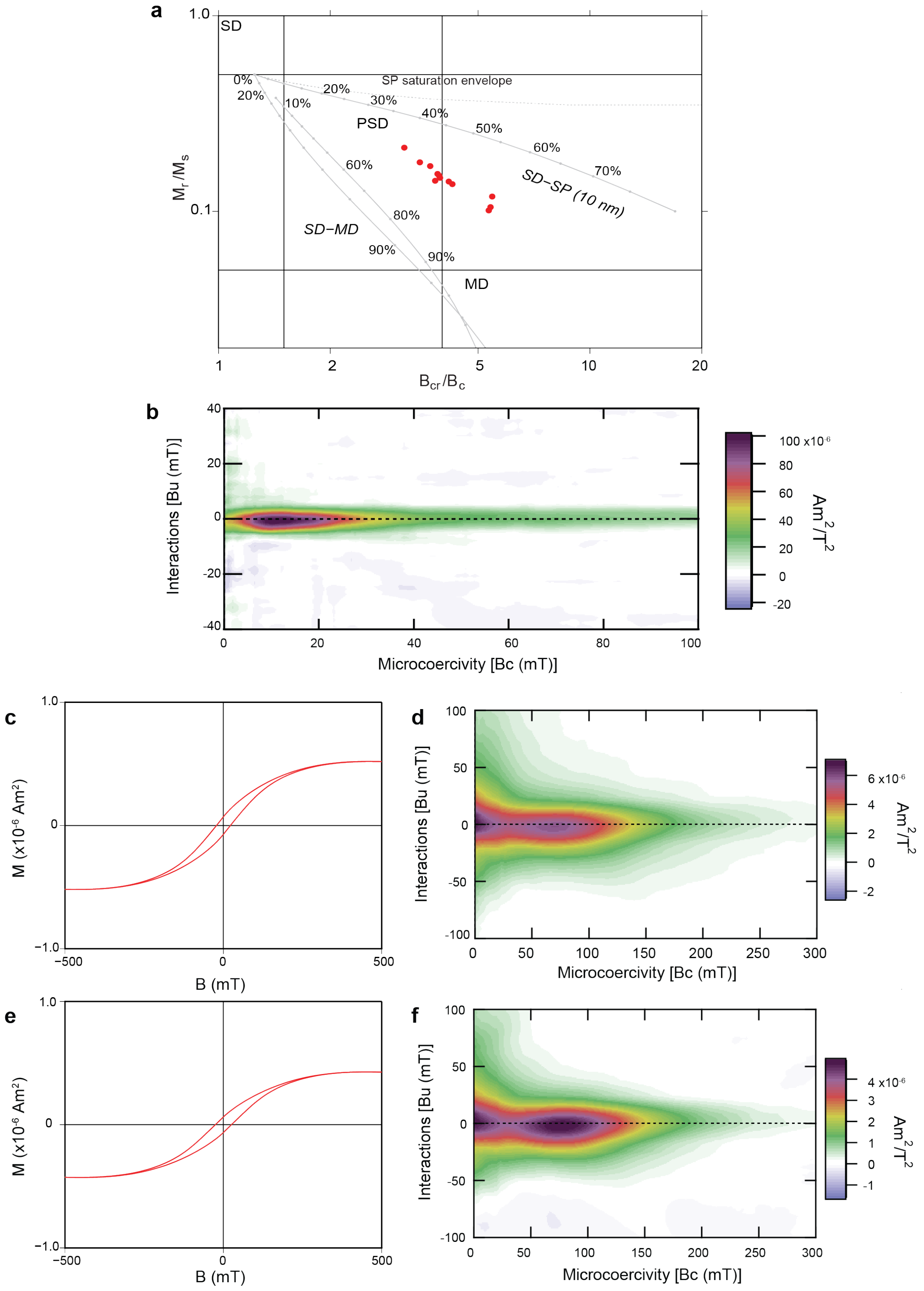

Supplementary Figure 1: **a**, Day Plot[36] of magnetic hysteresis parameters for Allende meteorite samples (see Methods). Values are derived from hysteresis loops measured to 0.5 T, corrected for paramagnetic slope (using 75-85% of the peak field). The mixing curves are for magnetite[37] and are shown only for reference. **b**, Example of FORC for ideal single domain magnetic carriers (magnetic needles in feldspars[38]). Saturating field, 10 T; number of FORCs, 100; Field increment, 1.839 mT; Smoothing criteria, Sc0, Sb0 = 7; Sc1, Sb1 = 8; Horiz. and Vert. λ = 0.1. FORCs were smoothed using FORCinel v3.01[39] and VARIFORC[40]. Hysteresis loops (**c, e**) measured using parameters described in (**a**); FORCs for these bulk Allende samples are shown in (**d, f**). These FORC measurements use a saturating field of 0.56 T, a field increment of 6.593 mT, with 100 FORCs. Smoothing criteria follow that shown in **b**. Note, all FORC plots are uncorrected for paramagnetic slope for best comparison with the literature.

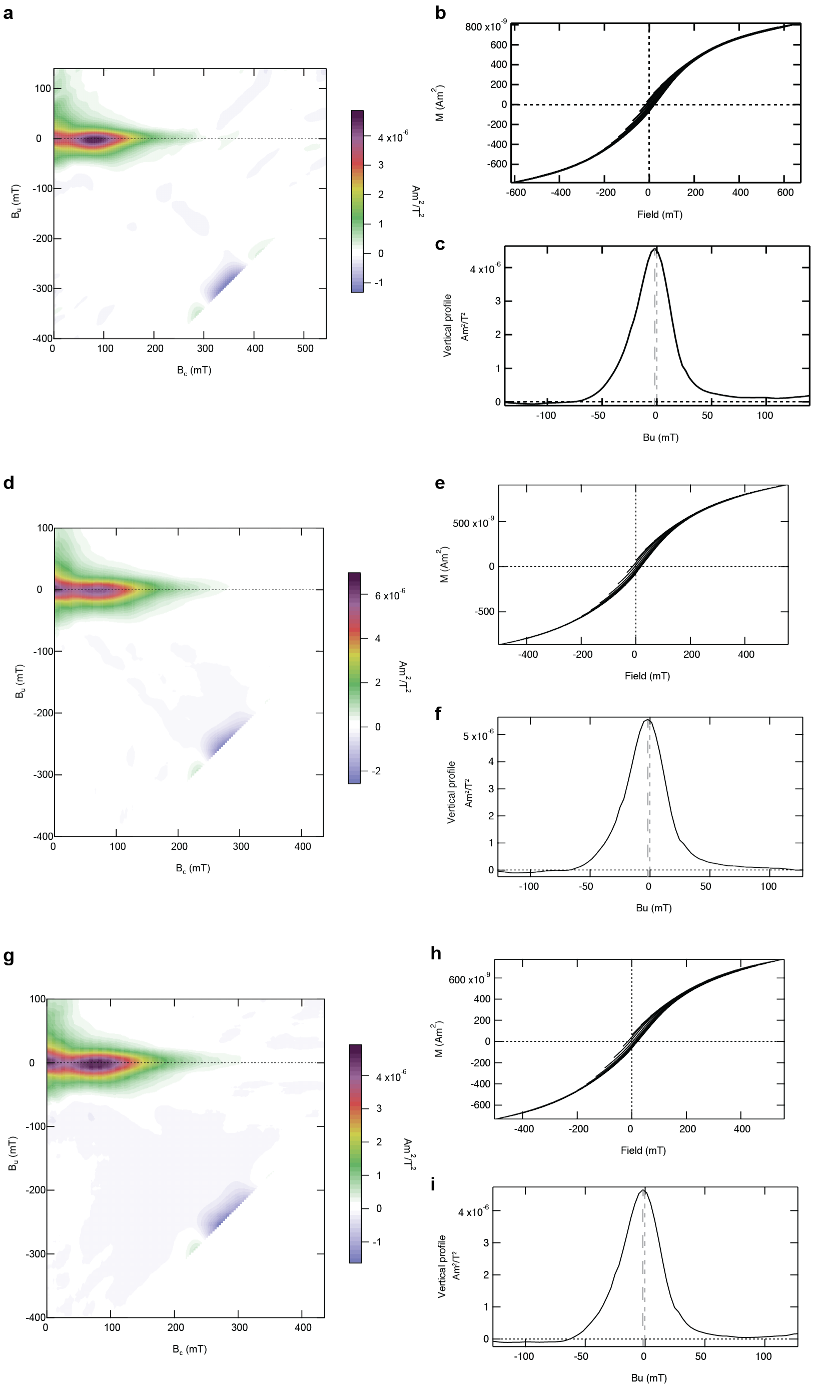

Supplementary Figure 2: **a**, Expanded FORC data example shown in Fig. 1b (saturating field, 0.7 T; number of FORCs, 125; field increment, 6.25 mT). These data lack the negative density region characteristic of SD grains. The following is the smoothing criteria for each FORC: Sc0, Sb0 = 7; Sc1 Sb1 = 8; Horiz. and Vert. $\lambda = 0.1$. **b**, Raw hysteresis data decimated to show every fifth FORC. **c**, Vertical profile of the FORC distribution taken at the peak. Dashed gray lines represent the peak of the vertical profile and the offset from zero. **d**, Expanded FORC data example shown in Supplementary Figure 1d. These data lack the negative density region characteristic of SD grains. **e**, Raw hysteresis data decimated to show every fifth FORC. **f**, Vertical profile of the FORC distribution taken at the peak. Dashed gray lines represent the peak of the vertical profile and the offset from zero. **g**, Expanded FORC data example shown in Supplementary Figure 1f. These data lack the negative density region characteristic of SD grains. **h**, Raw hysteresis data decimated to show every fifth FORC. **i**, Vertical profile of the FORC distribution taken at the peak. Dashed gray lines represent the peak of the vertical profile and the offset from zero.

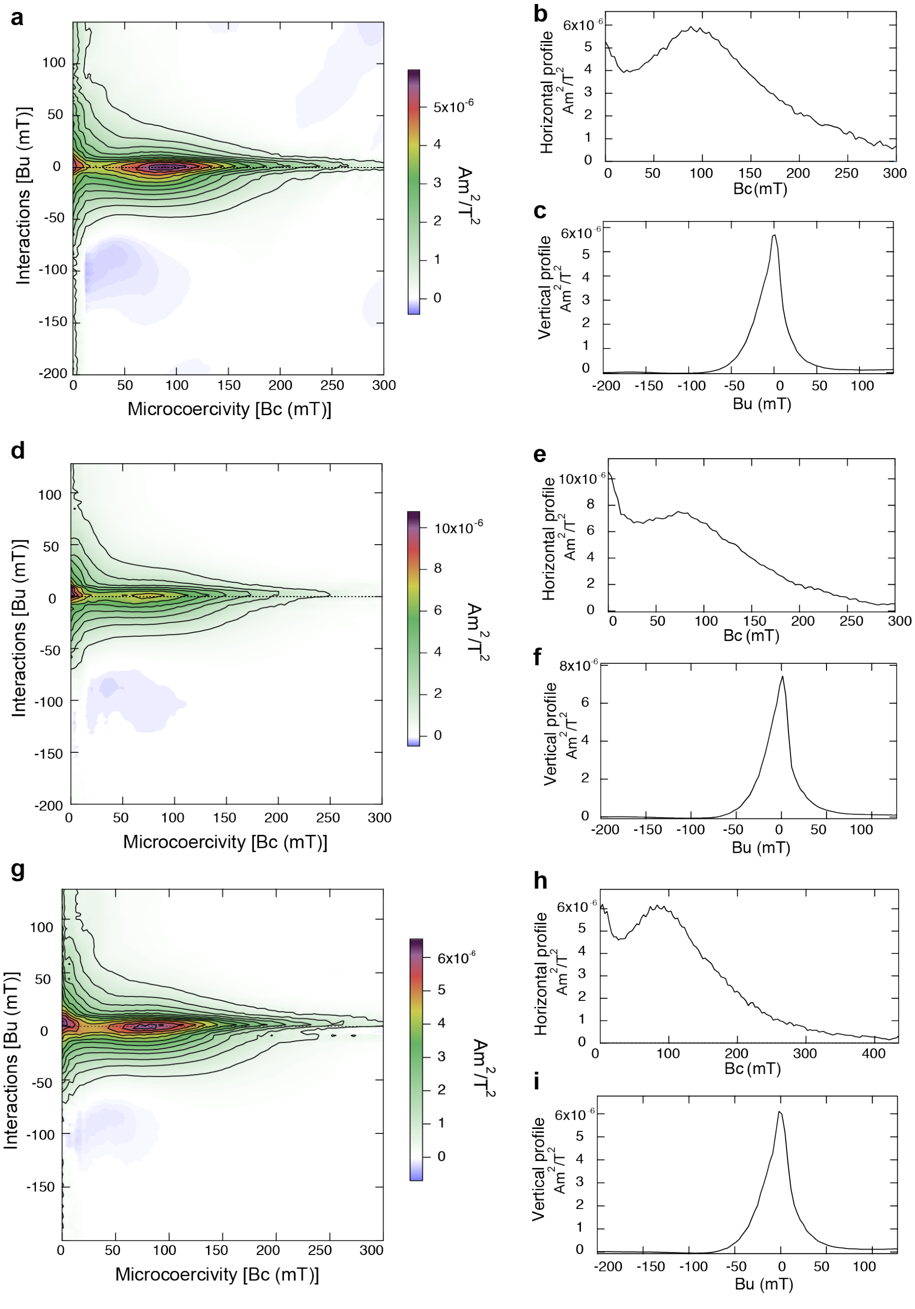

Supplementary Figure 3: a, d, g, Expanded FORC diagrams for examples shown in Fig. 1b, Supplementary Figure 1d, and Supplementary Figure 1f, respectively. The following is the variable smoothing parameters each FORC dataset: Sc0 = 4, Sb0 = 3; Sc1, Sb1 = 7; Horiz. and Vert. $\lambda = 0.3$. **b, e, h**, Horizontal profile of the respective FORC distribution taken at Bu = 0. **c, f, i,** Vertical profile of the respective FORC distribution taken at the peak (c, Bc = 95 mT; f, Bc = 75 mT; i, Bc = 80 mT).

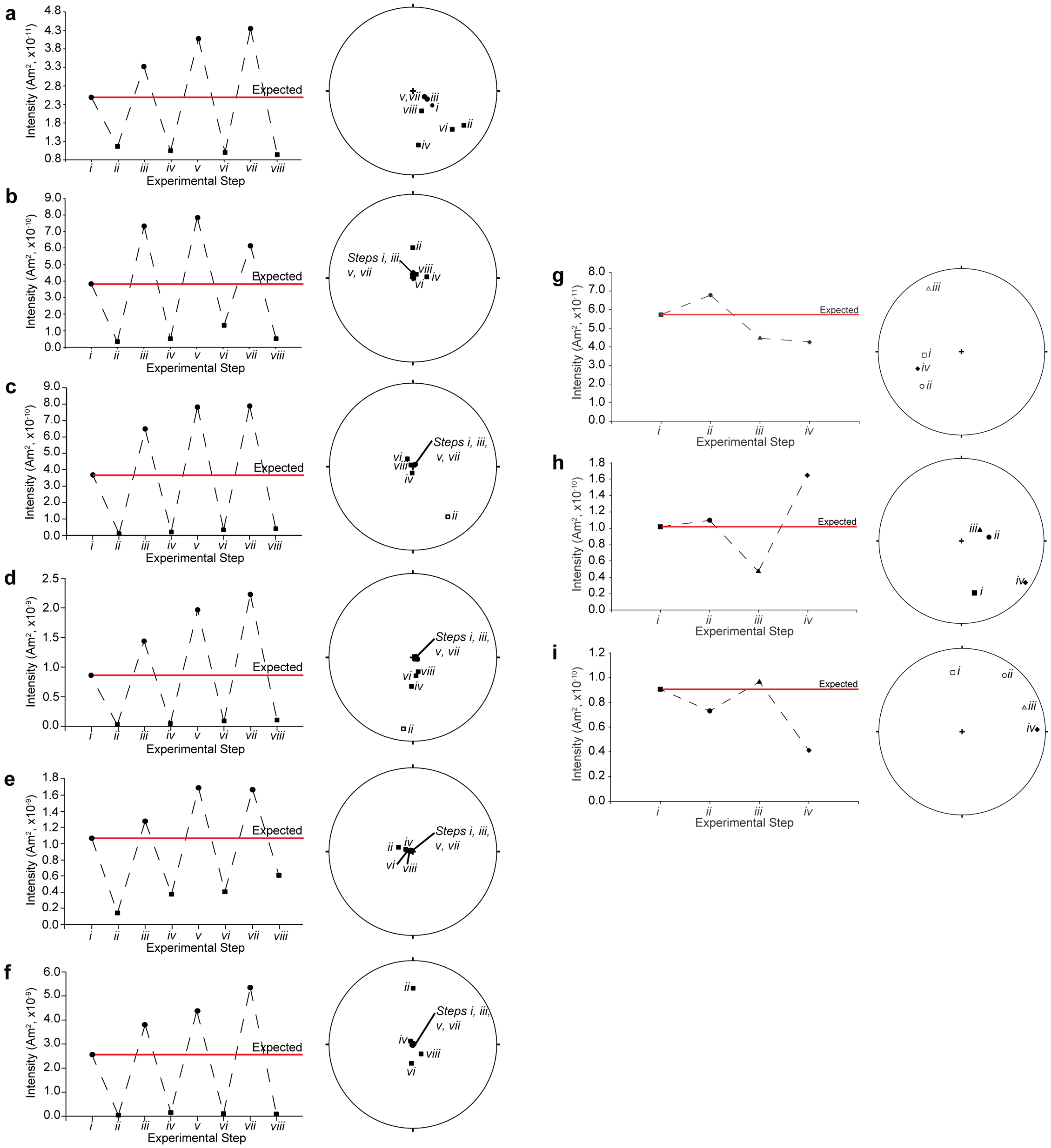

Supplementary Figure 4: a-f Cyclical heating experiments on Allende samples (see Methods) conducted in Nitrogen (**a, e**), Argon (**b-d**) and Air (**f**). Each experiment consisted of 4 repeats of the following cycle: 1) pTRM at ∼292 ^{o}C in a 30 μT field (black circle), 2) Demagnetization at ∼292 ^{o}C in zero field (black square). The expected intensity value for experiments is denoted by the labeled solid red line. The noise floor for these and other remanence measurements is ∼4-8 x 10^{-13} A m^2. **g-i**, Thermal pTRM directional experiments conducted in Ar atmosphere. Experimental Steps: 1) pTRM is imparted to the high susceptibility and the high Curie temperature magnetic carriers the sample (i.e. magnetite, awaruite) between 490 ^{o}C and 620 ^{o}C in a 30 μT field. Sample is cooled from 490 ^{o}C to room temperature in zero field (black square). 2) Sample is thermally demagnetized to 292 ^{o}C in zero field (black circle), 3) 340 ^{o}C in zero field (black triangle), and 4) 490 ^{o}C in zero field (black diamond). Red line denotes expected intensity values for samples after steps 2-4.

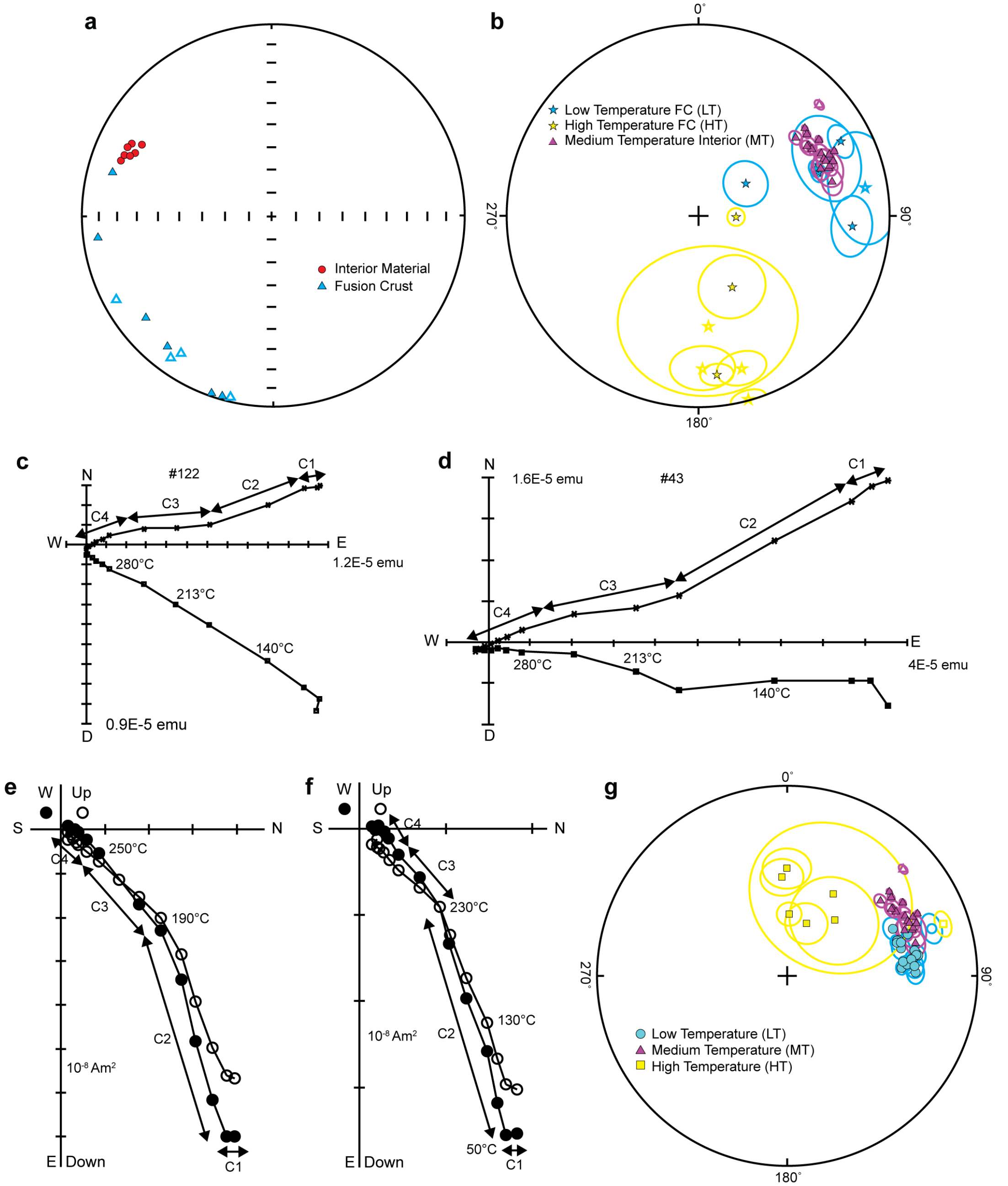

Supplementary Figure 5: **a**, Equal area stereonet comparing NRM values of interior samples (red circles) and exterior (fusion crust) samples (blue triangles). Solid symbols indicate lower hemisphere and open figures indicate upper hemisphere (after ref 11). Base panel reprinted from ref 11 with permission from Elsevier and modified as shown above. **b**, Equal area stereonet with data from ref 10 comparing interior MT directional component (red triangle) with LT and HT fusion crust sample components. **c-d**, Orthogonal vector plots of thermal demagnetization of bulk Allende samples (from ref 13). Note at least four components of remanence have been identified for each plot (C1-C4). Base panels reprinted from ref 13 with permission from J. Geophys. Res. and modified as above. **e-f**, Orthogonal vector plots of thermal demagnetization of bulk Allende samples (after ref 10). We identify four components of magnetization for the experiments (C1-C4). **g**, Summary stereonet of mean directions for LT, MT, and HT components from thermal demagnetization experiments of Allende. Filled and open symbols denote positive and negative inclinations respectively (after ref 10).

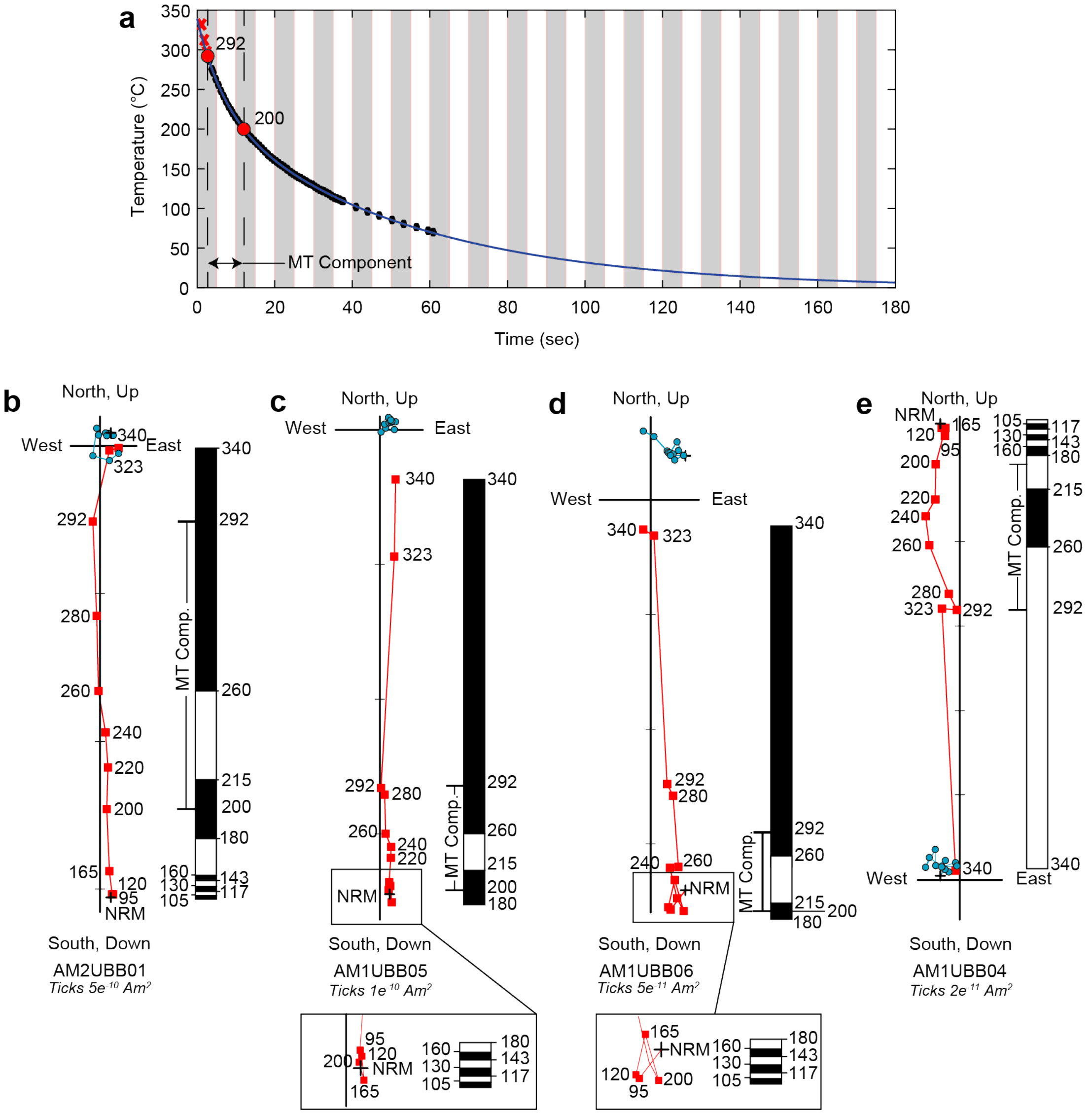

Supplementary Figure 6: **a**, Plot of calibration results obtained using an infrared pyrometer (black circles) with exponential cooling trends (solid blue lines). Red Xs on curves denote values that may indicate cooling by radiative heat transfer. The vertical dashed lines represent the time over which cooling occurs through the MT component of Allende (from ref 10). The red circles highlight the point of intersection of the dashed line on the calibration curve. **b-e**, Orthogonal vector plots for reversal experiments conducted in Ar with inclination (red squares) and declination (blue circles) plotted. Polarity scales are to the right of their respective vector plots denoting the anticipated field polarity at key temperature intervals (black and white denote lab induced polarity changes for reversal experiments).

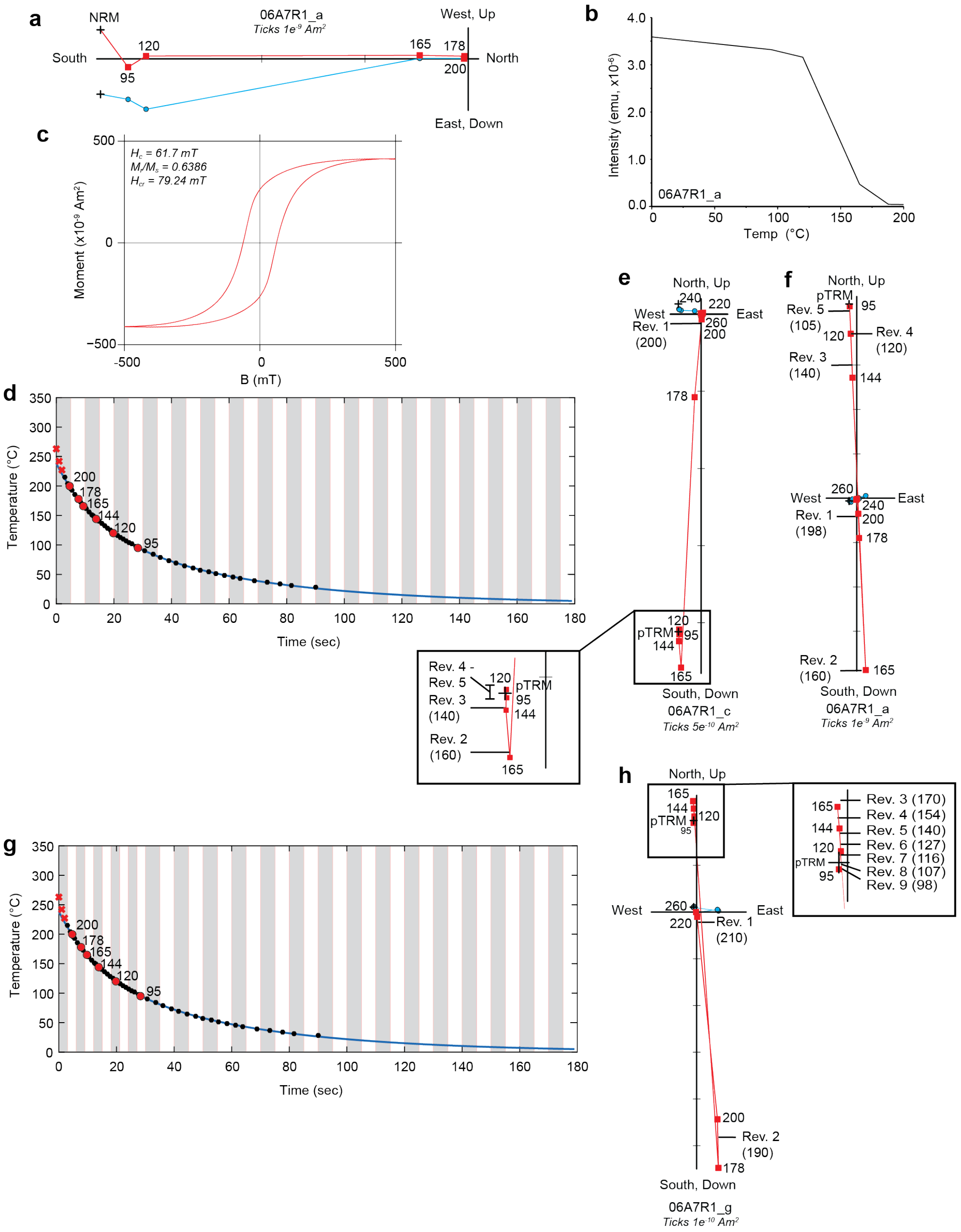

Supplementary Figure 7: **a**, Orthogonal vector plot of thermal demagnetization of terrestrial basalt, a typical titanomagnetite bearing sample from the Hawaiian-Emperor chain. **b**, Moment vs. temperature. **c**, Magnetic hysteresis curve, **d**, Calibration data (black circles) with exponential cooling trend (solid line). Red X's: values that may indicate cooling by radiative heat transfer. Five second alternating polarity intervals denoted by shaded and white vertical regions. The red circles highlight the location of the subsequent thermal demagnetization steps on the calibration curve. **e-f**, Orthogonal vector plots for CIRF experiments conducted in Ar with inclination (red squares) and declination (blue circles) plotted. Approximate temperatures at which applied field polarity was reversed are labeled on the orthogonal vector plot. **g**, Calibration with 3 second alternating polarity intervals for the first 30 seconds and 5 second alternating polarity intervals thereafter; intervals denoted by shaded and white regions. **h**, Orthogonal vector plot for CIRF conducted in Ar with inclination (red squares) and declination (blue circles) plotted. Approximate temperatures at which applied field polarity was reversed are labeled on the orthogonal vector plot.

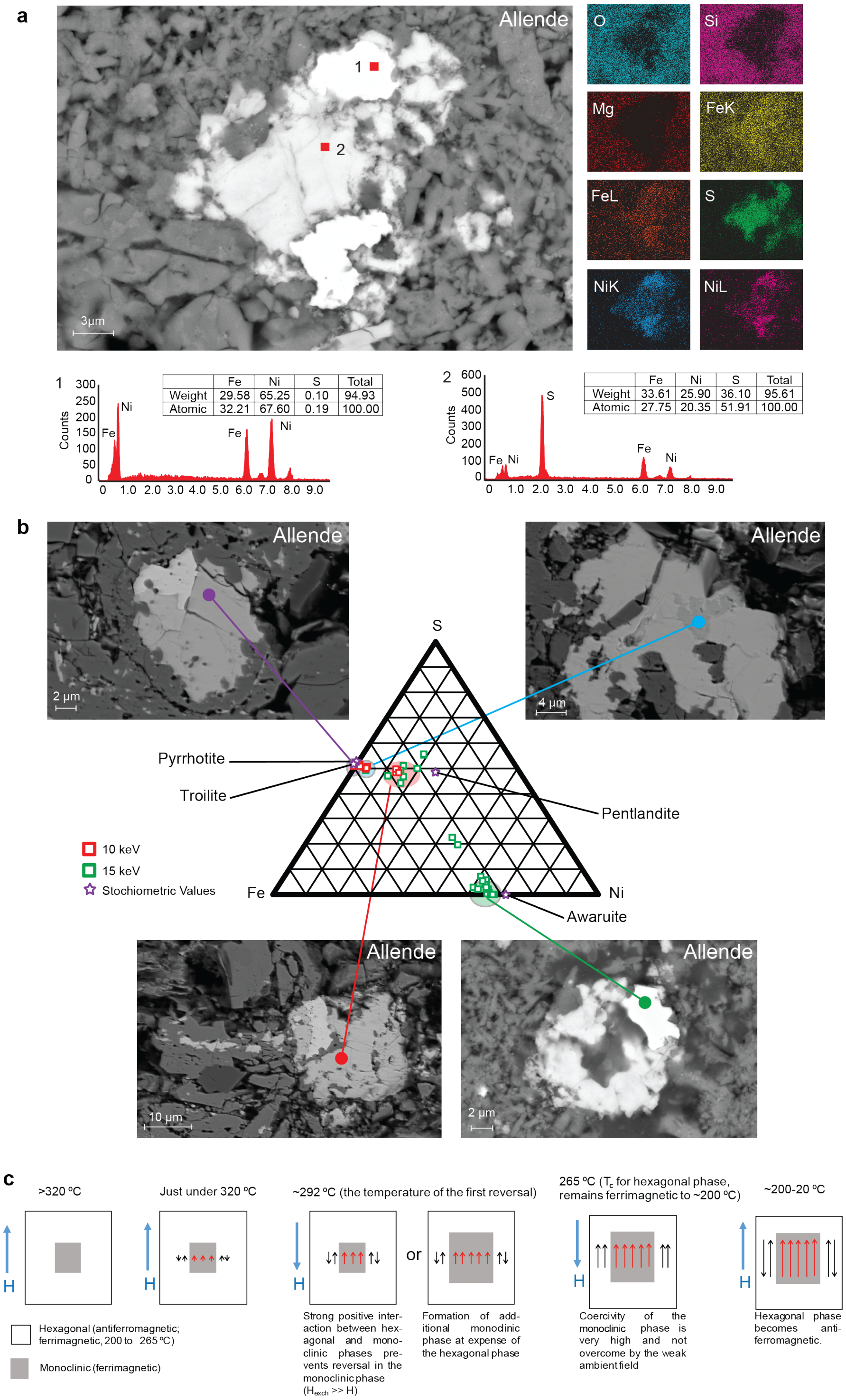

Supplementary Figure 8: **a**, Scanning electron microscope backscatter image (20 keV) with electron diffraction spectroscopy elemental maps, spectral and electron microprobe quantitative analyses. Numbered red squares correspond with numbered spectra below. Electron microprobe analyses shown in table inserts. **b**, Sulfur-Iron-Nickel ternary plot of sulfides and Ni-Fe grains from Allende microprobe analyses (Atomic %) with corresponding examples of each phase grouping. **c**, Preliminary interaction model for Allende. Magnetic moment (red arrows, length denotes relative moment) of a monoclinic pyrrhotite phase grows during cooling, and fails to record reversals due to exchange interactions (H_{exch}) and an intrinsic high coercivity, while the hexagonal phase progressively changes from an antiferromagnet, to a ferrimagnet and back to an antiferromagnet. Large H is applied magnetic field, which $\neq$ paleointensity due to interactions.

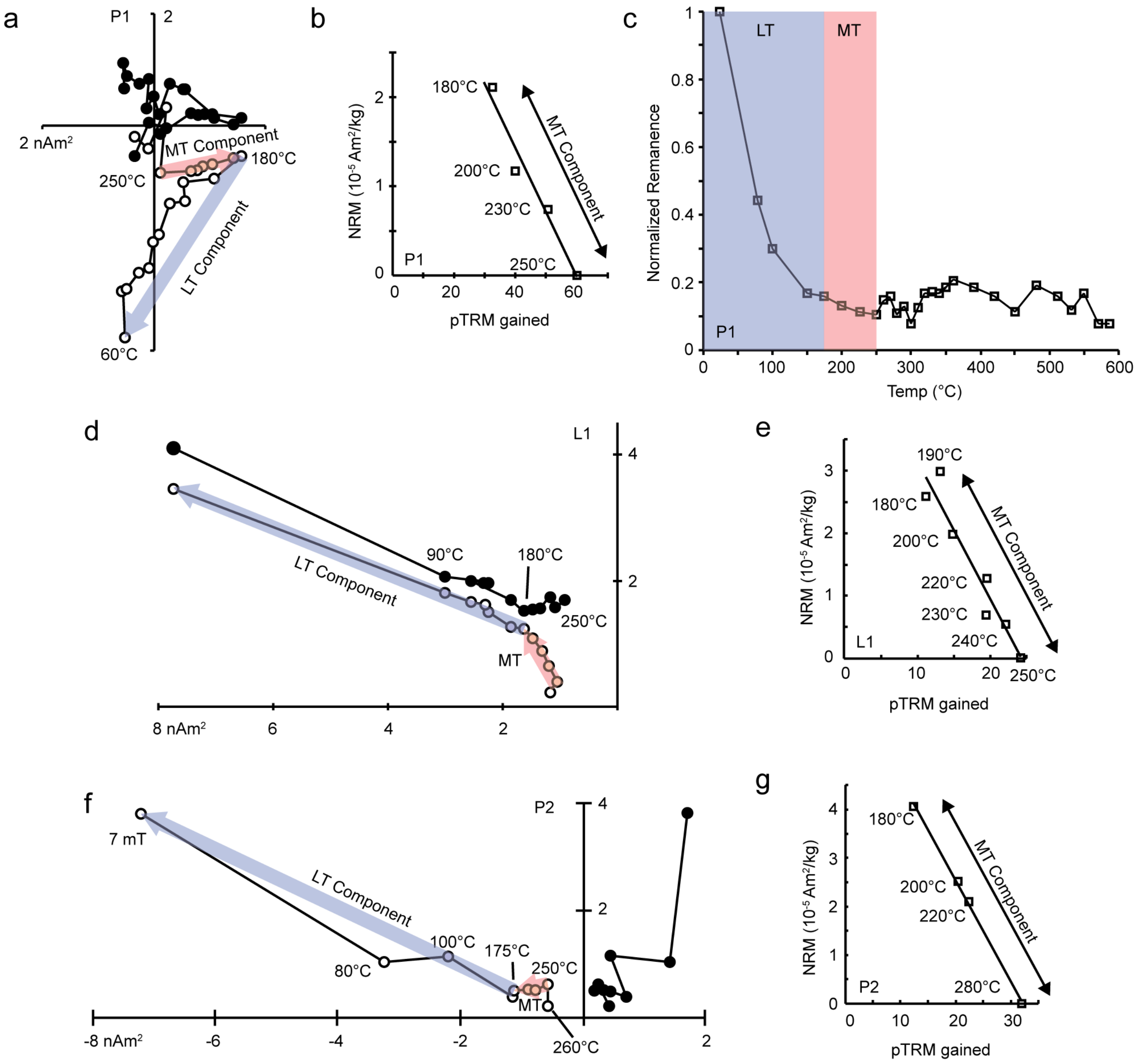

Supplementary Figure 9: Experimental results for Kaba sample P1: **a**, Orthogonal thermal demagnetization vector plot with LT (blue arrow) and MT (pink arrow) components denoted (**b**) Arai plot and (**c**) remanence vs. temperature plot with LT (blue) and MT (pink) temperature ranges shaded (from ref 23). The authors did not identify relative inclination and declination on their plots, but only state that they represent orthogonal components. For convenience of reference, we call open system "relative inclination" and closed symbols "relative declination". We interpret the "MT" component as quasi-linear; it does not trend to the origin and might contain multiple components. **d-e**, Orthogonal thermal demagnetization vector plots with LT (blue arrow) and MT (pink arrow) components denoted and Arai plots (applied field of 20 μT) for Kaba samples L1 and P2 (**f-g**) (from ref 23) interpreted here to be dominated by VRM. Base panels reprinted from ref. 23 with permission of Elsevier and modified as shown above.

Supplementary Table 1: Summary of published Allende paleointensity results*.

Sample Type	Experiment Type	Temp Range	Paleointensity (μT)	Ref.	Notes
Whole Rock	Thellier-Thellier	30-150 ˚C	131 ±42	*Butler* [11]	
			108 ±15		
			93 ±20		
		20 – 110 ˚C	123	*Banerjee & Hargraves* [41]	
			96		
		20 – 130 ˚C	111		
			106		
		Low Temp	~100	*Sugiura et al.* [42]	
		<130 – 330 ˚C	79 – 204	*Nagata & Funaki* [3]	
		0-230 ˚C (LT)	81.29 – 83.18	*Carporzen et al.* [10]	*Uncorrected*
			80.19 ±3.71		*Alteration Corrected*
		200-290 ˚C (MT)	109.34 – 136.24		*Uncorrected*
			59.01 ±14.13		*Alteration Corrected*
			~20		*Cooling Rate Corrected*
		>290 ˚C (HT)	2.2-3.66		*Uncorrected*
			3.61 ±0.76		*Alteration Corrected*
	AF – ARM Normalized		61.2 ±13.1	*Carporzen et al.* [10]	
	AF – IRM Normalized		55.3 ±15.5		
	NRM – ARM		73	*Nagata* [43]	
	Shashkanov-Metallova ARM		100 ±11	*Gus'Kova (Per. Comm, 1978) – In Nagata* [43]	
	PTRM-NRM (Non-Thermal)		95	*Brecher* [44]	
			25		
			15		
Matrix	Thellier-Thellier	<130 – 330 ˚C	190 – 290	*Nagata & Funaki* [3]	
Matrix, Bulk	Preisach		~6	*Muxworthy et al.* [45]	
Chondrules, Matrix, Bulk	REMc		13-60	*Emmerton et al.* [46]	
	Preisach		3-56		
Chondrules	Thellier-Thellier	160 – 300 ˚C	200-300	*Lanoix et al.* [47]	
		350 – 550 ˚C	1600		
		?	200-700	*Lanoix et al.* [48]	
	REMc		10.4 ±1.0	*Acton et al.* [49]	
CAI	Shaw Method		0.52-0.54	*Smethurst & Herrero-Bervera* [50]	

*The total range of paleointensity values is extraordinary relative to that of terrestrial rocks and minerals. The highest and lowest values can be partially traced to the component studied. The lowest values come from a CAI analysis. Other low values come from some chondrules and matrix (only those with estimates >290 ^{o}C). The highest values come from chondrites, whereas most of estimates from matrix are relatively strong. Although our focus is on the matrix and temperature range interpreted in prior studies to represent a primary magnetization (<290 ^{o}C), we believe the profound interactions observed and the complex magnetic mineralogy question the veracity of any of these values as true paleointensity measures.

Additional information on magnetic measurement units can be found in the following sources: Shive, P.N. Suggestions for the use of SI units in magnetism, *Eos Trans. AGU*, **67**, 25 (1986); Butler, R.F. *Paleomagnetism: Magnetic Domains to Geologic Terranes*, Blackwell Scientific Publications, Boston, 336 p. (1992).

Supplementary Movie 1: Select magnetic field lines drape over the surface of the asteroid (shown in transparent gray). The field lines are colored by their strength relative to the incoming solar wind values. The field lines were integrated from points evenly spaced along a line across the bottom boundary ($y = -8\, r_{\mathrm{asteroid}}$ & $z = 0$), as well as from points within a circle of radius $3\, r_{\mathrm{asteroid}}$ centered at the center of the asteroid. The slice inside the asteroid shows the azimuthally symmetric resistivity structure. The asteroid is stationary to highlight the field amplification. To see how this relates to magnetization acquisition for a rotating asteroid, see "Parent body rotation" in this Supplementary Information document.

References

1. Flores-Gutiérrez, D., Urrutia-Fucugauchi, J. Hysteresis properties of chondritic meteorites: New results for chondrules from the Allende meteorite. *Geofis Int* **41**, 179-188 (2002).
2. Thorpe, A.N., Senftle, F.E., Grant, J.R. Magnetic study of magnetite in the Tagish Lake meteorite. *Meteorit Planet Sci* **37**, 763-771 (2002).
3. Nagata, T., Funaki, M. Paleointensity of the Allende carbonaceous chondrites. *Mem Natl Inst Polar Res Spec Issue* **30**, 403-434 (1983).
4. Wasilewski, P. New magnetic results from Allende C3 (V). *Phys Earth Planet Inter* **26**, 134-148 (1981).
5. Verwey, E.J. Electronic conduction of magnetite (Fe_3O_4) and its transition point at low temperature. *Nature* **144**, 327-328 (1939).
6. Macke, R.J., Consolmagno, G.J., Britt, D.T. Density, porosity, and magnetic susceptibility of carbonaceous chondrites. *Meteor Planet Sci* **46**, 1842-1862 (2011).
7. Dunlop, D.J., Özdemir, Ö. *Rock magnetism, Fundamentals and Frontiers* (Cambridge Univ. Press, Cambridge, UK, 1997).
8. Dekkers, M.J. Magnetic properties of natural pyrrhotite. II. High-and low-temperature behaviour of Jrs and TRM as function of grain size. *Phys Earth Planet Inter* **57**, 266-283 (1989).
9. Wasilewski, P.J., Saralker, C. Stable NRM and mineralogy in Allende: Chrondrules. *Proc. Lunar Planet. Sci.* **12B**, 1217-1227 (1981).
10. Carporzen, L. et al. Magnetic evidence for a partially differentiated carbonaceous chondrite parent body. *Proc Natl Acad Sci USA* **108**, 6386-6389 (2011).
11. Butler, R.K. Natural remanent magnetization and thermomagnetic properties of the Allende meteorite. *Earth Planet Sci Lett* **17**, 120-128 (1972).
12. Fu, R.R., Lima, E.A., Weiss, B.P. No nebular magnetization in the Allende CV carbonaceous chondrite. *Earth Planet Sci Lett* **404**, 54-66 (2014).
13. Sugiura, N., Strangway, D.W. NRM directions around a centimeter sized dark inclusion in Allende. *J. Geophys Res Suppl* **90**, c729-c738 (1985).
14. Lévesque, L. Law of cooling, heat conduction and Stefan-Boltzmann radiation laws fitted to experimental data for bones irradiated by CO_2 laser. *Biomed Opt Exp* **5**, 701-712 (2014).
15. Tarduno, J.A., Duncan, R.A., Scholl, D.W. Shipboard Scientific Party, Proc. ODP, Init. Repts. (Ocean Drilling Program, College Station, TX), **197** (2002).
16. Tarduno, J.A., et al., The Emperor Seamounts: Southward motion of the Hawaiian Hotspot plume in Earth's mantle, *Science* **301**, 1064-1069 (2003).
17. Clarke Jr., R.S. The Allende, Mexico, meteorite shower. *Smithson Contr Earth Sci* **5**, 1-53 (1971).
18. Vaughan, D.J., Schwarz, E.J., Owens, D.R. Pyrrhotites from the Strathcona Mine, Sudbury, Canada; A thermomagnetic and mineralogical study. *Econ Geol* **66**, 1131-1144 (1971).
19. Sztrókay, K.I., Tolnay, V., Foldvari-Vogl, M. Mineralogical and chemical properties of the carbonaceous meteorite from Kaba, Hungary. *Acta Geol* (Hungary) **7**, 57-103 (1961).
20. Rubin, A.E., Grossman, J.N. Phosphate-sulfide assemblages and Al/Ca rations in type-3 chondrites. *Meteoritics* **20**, 479-489 (1985).
21. Keller, L.P., Buseck, P.R. Aqueous alteration in the Kaba CV3 carbonaceous chondrite. *Geochim Cosmochim Acta* **54**, 2113-2120 (1990).
22. Hua, X., Huss, G.R., Tachibana, S., Sharp, T.G. Oxygen, silicon, and Mn-Cr isotopes of fayalite in the Kaba oxidized CV3 chondrite: Constraints for its formation history. *Geochim Cosmochim Acta* **69**, 1333-1348 (2005).

23. Gattacceca, J., Weiss, B.P., Gounelle, M. New constraints on the magnetic history of the CV parent body and the solar nebula from the Kaba meteorite. *Earth Planet Sci Lett* **455**, 166-175 (2016).
24. Watson, D.E., Larson, E.E., Herndon, J.M., Rowe, M.W. Thermomagnetic analysis of meteorite, 2. C2 chondrites. *Earth Planet Sci Lett* **27**, 101-107 (1975).
25. Krot, A.N. et al. Progressive alteration in CV3 chondrites: More evidence for asteroidal alteration. *Meteor Planet Sci* **33**, 1065-1085 (1998).
26. Baumgartner, J. et al. Nucleation and growth of magnetite from solution. *Nature Materials* **12**, 310-314 (2013).
27. Cournede, C. et al. An early solar system magnetic field recorded in CM chondrites. *Earth Planet Sci Lett* **410**, 62-74 (2015).
28. Kletetschka, G., Kohout, T., Wasilewski, P.J. Magnetic remanence in the Murchison meteorite. *Meteorit Planet Sci* **38**, 399-405 (2003).
29. Brownlee, D.E. Comets. in *Meteorites, Comets, and Planets: Treatise on Geochemistry*, Second Edition, Volume 1 (eds Davis, A.M.) 663-737 (Elsevier Science & Technology) (2005).
30. Tarduno, J.A., OBrien, T.M., Blackman, E.G., Smirnov, A.V. Magnetization of CV meteorites in the absence of a parent body core dynamo, *Lunar Planet Sci Conf* **XLVIII**, 2850 (2017).
31. Oran, R., Weiss, B.P., Cohen, O. Were chondrites magnetized by the early solar wind? *Earth Planet Sci Lett* **492**, 222-231 (2018).
32. Duba, A. G., Boland, J. N. High Temperature Electrical Conductivity of the Carbonaceous Chondrites Allende and Murchison. *Lunar and Planetary Science Conference* **XV**, 232-233 (1984).
33. Owens, M.J., Forsyth, R.J. The heliospheric magnetic field. *Living Rev. Sol. Phys.* **0**,5. https://doi.org/10.12942/lrsp-2013-5 (2013).
34. See, V. et al. The connection between stellar activity cycles and magnetic field topology. *Mon Not Roy Astron Soc* **462**, 4442-4450 (2016).
35. Rubincam, D.P. Radiative spin-up and spin-down of small asteroids. *Icarus* **148**, 2-11 (2000).
36. Day, R., Fuller, M., Schmidt, V.A. Hysteresis properties of titanomagnetites: grain-size and compositional dependence. *Phys Earth Planet Inter* **13**, 260-267 (1977).
37. Dunlop, D.J. Theory and application of the Day plot (Mrs/Ms versus Hcr/Hc) 1. Theoretical curves and tests using titanomagnetite data. *J Geophys Res* **107**, B32056 (2002).
38. Bono, R.K., Tarduno, J.A. A stable Ediacaran Earth recorded by single silicate crystals of the ca. 565 Ma Sept-Îles intrusion. *Geology* **43**, 131-134 (2015).
39. Harrison, R.J., Feinberg, J.M. FORCinel: An improved algorithm for calculating first-order reversal curve distributions using locally weighted regression smoothing. *Geochem Geophys Geosyst* **9**, Q05016 (2008).
40. Egli, R. VARIFORC: an optimized protocol for the calculation of non-regular first-order reversal curve (FORC) diagrams. *Global Planet Change* **110**, 302-320 (2013).
41. Banerjee, S.K., Hargraves, R.B. Natural remanent magnetizations of carbonaceous chondrites and the magnetic field in the early solar system. *Earth Planet Sci Lett* **17**, 110-119 (1972).
42. Sugiura, N., Lanoix, M., Strangway, D.W., Magnetic fields of the solar nebula as recorded in chondrules from the Allende meteorite. *Phys Earth Planet Inter* **20**, 342-349 (1979).
43. Nagata, T. Meteorite magnetism and the early solar system magnetic field. *Phys Earth Planet Inter* **20**, 324-341 (1979).
44. Brecher, A. Meteoric Magnetism: Implications for parent bodies of origin. In *Comets, Asteroids and Meteorites* (ed. Delsemme, A.H.) 415-427 (Univ. Toledo Press, Toledo, OH USA, 1977).
45. Muxworthy, A.R. et al. Evidence for an impact-induced magnetic fabric in Allende, and exogenous alternatives to the core dynamo theory for Allende magnetization. *Meteoritics Planet Sci* **52**, 2132-2146 (2017).
46. Emmerton, S., Muxworthy, A.R., Hezel, D.C., Bland, P.A. Magnetic characteristics of CV chondrules with paleointensity implications. *J Geophys Res* **116**, E12007 (2011).
47. Lanoix, M., Strangway, D.W., Pearce, G.W. The primordial magnetic field preserved in chondrules of the Allende meteorite. *Geophys Res Lett* **5**, 73-76 (1978).
48. Lanoix, M., Strangway, D.W., Pearce, G.W. Paleointensity determinations from Allende Chondrules. *Lunar Planet Sci Conf* **IX**, 630-632 (1978).

49. Acton, G. et al. Micromagnetic coercivity distributions and interactions in chondrules with implications for paleointensities of the early solar system. *J Geophys Res* **112**, B03S90 (2007).
50. Smethurst, M.T., Herrero-Bervera, E. Paleomagnetic Analysis of Calcium-Aluminum Inclusions (CAI's) from the Allende Meteorite. AGU Fall Meeting Abstracts, GP72A-0989 (2002).
